# Annotation of the *Turnera subulata* (Passifloraceae) Draft Genome Reveals the *S*-Locus Evolved after the Divergence of Turneroideae from Passifloroideae in a Stepwise Manner

**DOI:** 10.3390/plants12020286

**Published:** 2023-01-07

**Authors:** Paige M. Henning, Eric H. Roalson, Wali Mir, Andrew G. McCubbin, Joel S. Shore

**Affiliations:** 1 School of Biological Sciences, Washington State University, Pullman, WA 99164-4236, USA; 2 Center for Genomic Science Innovation, University of Wisconsin Madison, 425 Henry Mall, Madison, WI 53706-1577, USA; 3 Department of Biology, York University, 4700 Keele Street, Toronto, ON M3J 1P3, Canada

**Keywords:** genome, *Turnera subulata*, distyly, heterostyly, *YUCCA*, *S-protein homolog*, *BAHD*

## Abstract

A majority of *Turnera* species (Passifloraceae) exhibit distyly, a reproductive system involving both self-incompatibility and reciprocal herkogamy. This system differs from self-incompatibility in *Passiflora* species. The genetic basis of distyly in *Turnera* is a supergene, restricted to the *S*-morph, and containing three *S*-genes. How supergenes and distyly evolved in *Turnera*, and the other Angiosperm families exhibiting distyly remain largely unknown. Unraveling the evolutionary origins in *Turnera* requires the generation of genomic resources and extensive phylogenetic analyses. Here, we present the annotated draft genome of the S-morph of distylous *Turnera subulata*. Our annotation allowed for phylogenetic analyses of the three *S*-genes’ families across 56 plant species ranging from non-seed plants to eudicots. In addition to the phylogenetic analysis, we identified the three *S*-genes’ closest paralogs in two species of *Passiflora*. Our analyses suggest that the *S*-locus evolved after the divergence of *Passiflora* and *Turnera*. Finally, to provide insights into the neofunctionalization of the *S*-genes, we compared expression patterns of the *S*-genes with close paralogs in *Arabidopsis* and *Populus trichocarpa*. The annotation of the *T. subulata* genome will provide a useful resource for future comparative work. Additionally, this work has provided insights into the convergent nature of distyly and the origin of supergenes.

## 1. Introduction

**Figure 1:**
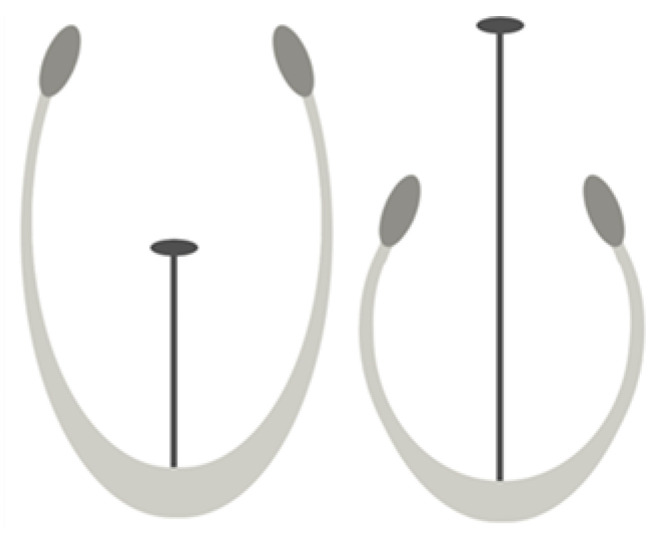
Representative diagram of the S-morph (left) and L-morph (right) in distylous *Turnera*.

Distyly, a form of heteromorphic self-incompatibility, is a reproductive system found in 28 genera of angiosperms [1]. Distylous populations contain two floral morphs, the S-morph and L-morph, showing morphological differences (Figure 1) accompanied by self- (and intra-morph) incompatibility (SI). The genetic basis of these characteristics is a supergene called the *S*-locus; the *S*-morph is hemizygous for the *S*-locus in *Primula* [2], *Turnera* [3], *Linum* [4], and likely in *Fagopyrum* [5]. Despite its complex phenotype, distyly has evolved across 28 genera with extreme phenotypic consistency [1], making it a remarkable example of convergent evolution.

The *S*-locus has been characterized in *Primula* [2,6,7,8], *Turnera* [3], and *Linum* [4]. The *S*-locus in *Primula* contains five *S*-genes [2], two of which have been characterized. *GLO* is involved in elongation of the corolla below the stamen, leading to differential anther positioning between morphs [8]. *CYP734A50* determines style length and female mating type via inactivation of brassinosteroids (BR) [9,10]. Recent phylogenetic analyses of Primula’s five *S*-genes suggest that four arose via independent duplication events followed by translocation to the pre-*S*-locus [11].

In *Linum,* the *S*-locus contains nine hypothetical *S*-genes, five with unknown functions, a homolog of *AtVUP1* named *TSS1*, *WDR-44*, a tetratricopeptide repeat-containing protein, and a gene likely involved in pollen development [4]. Only five of the genes are expressed, suggesting that the others may be nonfunctional. *TSS1* is likely involved in style length dimorphism [4,12]. Positioning of the hypothetical *S*-genes’ paralogs within the *Linum* genome suggests, similar to *Primula*, that the genes were acquired in several independent duplication events.

In *Turnera,* the *S*-locus is composed of three *S*-genes, *BAHD*, *SPH1*, and *YUC6* [3]. *BAHD*, a member of the *BAHD* acyltransferase family, determines style length and female mating-type via inactivation of BR [13,14]. *SPH1*, a member of the *S*-*protein homolog* family, likely controls filament length [3]. *YUC6*, a member of the *YUCCA* flavin monooxygenase family, controls male mating-type [15] likely via alterations of auxin concentration in the S-morph relative to the L-morph [16]. Currently, it is unknown how the *S*-locus evolved in *Turnera*.

Analysis of *S*-loci across heterostylous genera has revealed similarities at the metabolic and genetic levels. The basis of style length and female mating type in both *Primula* and *Turnera* is inactivation of BR. Indirect alterations to the Phytochrome Interacting Factor (PIF) signaling hubs via inactivation of BR or direct alteration of PIF signaling hub-related genes may be the basis of repression of style elongation in *Fagopyrum*, *Primula,* and *Turnera* [16], a hypothesis that has been computationally supported [11]. Auxin appears to play a role in stamen elongation in *Fagopyrum* [11], potentially similar to filament elongation in *Turnera*, as suggested by computational analysis [16]. The commonality in the evolution of the *S*-locus in *Linum* and *Primula* suggests a further convergent nature at the genomic level. In other systems displaying convergent evolution, there is often a convergent genetic basis in addition to the convergent phenotype [17,18,19]. Therefore, one might predict mechanistic convergence at the genomic level in distylous species via hemizygosity, as appears to be emerging from studies of *Primula* and *Linum.*

To further understand the convergent nature of distyly, evolution of supergenes, and neofunctionalization of the *S*-genes, genomic and phylogenetic analyses of different genera exhibiting distyly is necessary. Here we present an annotation of the *Turnera subulata* (2x) genome [3]. Using this genomic resource, we identified the closest paralogs of the three *S*-genes. The genomic locations of these paralogs suggest that the three *S*-genes underwent independent duplication events. We used phylogenetic analyses to estimate when the *S*-genes underwent their individual duplications to the pre-*S*-locus. Our analyses suggest that these duplication events occurred after the divergence of the subfamily Turneroideae from Passifloroideae. These results support the previously proposed hypotheses that the convergent nature of distyly does not just occur at the phenotypic and metabolic level, but also at the molecular level, involving independent acquisition of the *S*-genes to the hemizygous *S*-locus.

## 2. Results

### 2.1. Genome Assembly and Annotation

The genome assembly consists of 7877 scaffolds. After filtering and manual curation, a total of 30,992 genes were annotated. It was predicted that 979 genes encode tRNAs and 29,943 encode proteins. BUSCO (v.5.4.3) [20] was used to assess the genome assembly and annotation. We detected 96.6% of the core eukaryotic genes in the gDNA assembly and 87.9% in the annotated CDS (based on homology to *A. thaliana*). We had also previously sequenced bacterial artificial chromosome clone contigs of the *S*-locus haplotypes [3]. Our final protein-coding gene count (29,976) is similar to that of *Ricinus communis* (28,584 v0.1) [21] and *Theobroma cacao* (27,379 v2.1) [22], but smaller than *Manihot esculenta* (32,858 v8.1) [23] and *Populus trichocarpa* (34,699 v4.1) [24]. Interestingly, this final count is greater than both *Passiflora edulis* (23,171) and *Passiflora organensis* (25,327), members of the same family.

**Figure 2:**
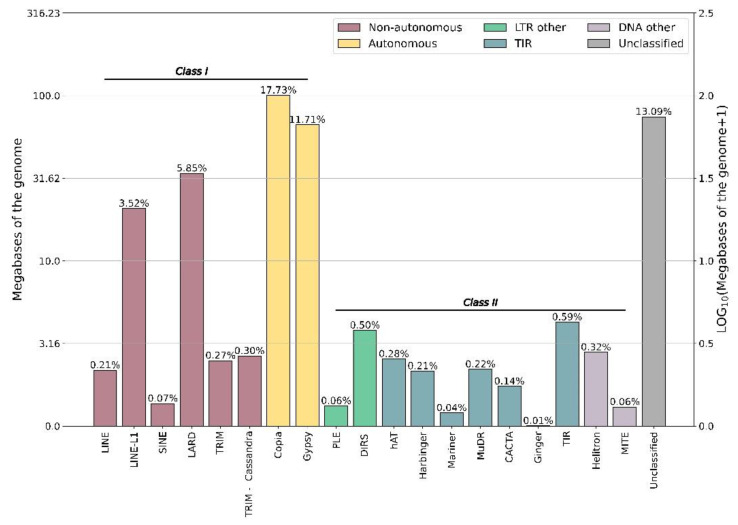
Repetitive elements (RE) identified in the *Turnera subulata* genome. Percentages represent the percent of each kind of RE. The graph is scaled to the log_10_(Megabases + 1) and shows the extent of the genome occupied by each element type.

Our analysis revealed that 53.63% of the *T. subulata* genome is composed of repetitive elements (Figure 2); this is somewhat less than *Passiflora*. The *P. organensis* genome is composed of 58.55% repetitive elements [25], *P. cincinnata* (60.49%) and *P. quadrangularis* (73.42%) [26]. We also analyzed the repetitive element composition of the *S*-locus haplotypes using EDTA [27] (Supplementary Figure S1). The “recessive” *s*-haplotype is 324,544 bp in length, containing 122 repetitive elements (69 Class I; 53 Class II) that comprise 25.06% of the length of the haplotype. In stark contrast, the considerably longer “dominant” *S*-haplotype is 1,560,574 bp in length, containing 1040 repetitive elements (843 Class I; 197 Class II) that comprise 57.35% of the sequence. The dominant haplotype also contains a number of helitron elements (26) that are absent from the recessive, as well as unclassified LTR elements.

The rearrangements of the dominant haplotype are expected to lead to recombination suppression in the regions flanking the *S*-locus (Supplementary Figure S1). The accumulation of repetitive elements and synonymous mutations are expected in regions of suppressed recombination. We estimated synonymous substitution rates (Ks) of the genes flanking the *S*-locus between haplotypes to test this expectation. Genes within the “rearranged/inverted” region should have higher Ks than those outside of the inversions (Supplementary Figure S2). The full extent of rearrangement of the dominant vs. the recessive haplotypes are not known with certainty, particularly at the upstream end (*AP2* gene).

We found that substitution rates varied across the haplotypes, ranging from 0.004 at *CYCLIN D* (*CYCD*) to 0.140 at *AP2*. Collinearity of the two haplotypes is exhibited downstream (genes *CYCD*, *ADPRF*, and *EB1C*), where we might expect recombination to occur. A comparison of Ks for genes anticipated to be undergoing recombination vs. the suppressed genes gives genes *CYCD*-*EB1* mean Ks = 0.012 vs. genes *AP2*—*SUMO* Ks = 0.061 (t19 = 2.50, *p* < 0.02), providing evidence for recombination suppression for genes within the rearranged portion of the haplotypes.

### 2.2. Comparison of Orthologous Groups between Members of Malpighiales

**Figure 3:**
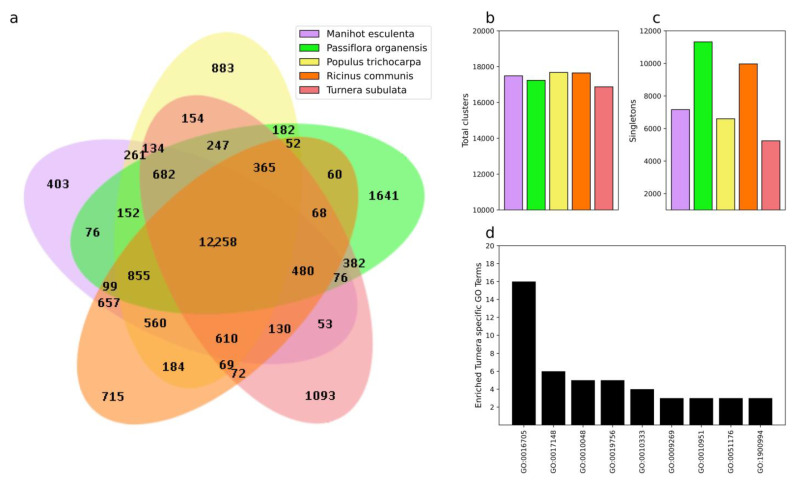
OrthoVenn2 results of the Malpighiales comparison. Venn diagram showing orthologous groups shared between various combinations of the five species as well as proteins that were specific to a single species (**a**). Total number of clusters for each species including both orthologous and paralogous clusters (**b**). Total singletons for each species; these represent proteins that are predicted to not share a similar function with other orthologs or paralogs (**c**). Enriched GO terms for *Turnera* specific paralogous groups (**d**).

OrthoVenn2 was used to identify orthologous groups among *T. subulata* and *P. organesis* (Supplementary Figure S3) and other members of Malpighiales (Figure 3). Identification of orthologous groups helped confirm the quality of annotation while simultaneously identifying potential neofunctionalization and enriched GO terms specific to a species. In our Turneroideae and Passifloroideae comparison, we identified 1285 orthologous groups specific to *Turnera* with 16 enriched GO terms (Supplementary Figure S3a,b). *Turnera* has fewer “singletons” (5759) or genes without paralogous/orthologous groups than *P. organensis* (7543) but more than *P. edulis* (Supplementary Figure S3c). Two of the highest enriched GO terms associated with *Turnera* were GO:0016705 “oxidoreductase activity acting on paired donors with incorporation or reduction of molecular oxygen” and GO:0006952 “defense response” (Supplementary Figure S3d). Interestingly, four groups were enriched for GO:0060320 “rejection of self-pollen”.

In our comparison of the Malpighiales, we observed a reduction in *T. subulata-*specific paralogous groups (from 1285 to 1093, a decrease of 192); approximately the same reduction was observed in *P. organensis* (from 1832 to 1641, a decrease of 191) (Figure 3a). Additionally, we also observed a drop in GO terms (16 to 9). Interestingly, GO:0016705 remained the top enriched GO term (Figure 3b,d), suggesting that genes found in these groups are potentially important for a unique feature of *T. subulata*. *T. subulata* showed the smallest number of singletons (5759 to 5245) (Figure 3c). *TsYUC6* does not fall into an orthologous group and is labeled as a singleton, *TsBAHD* falls into an orthologous group with eight other proteins associated with alkaloid metabolism, and *TsSPH1* falls into a paralogous group related to rejection of self-pollen (Supplementary Table S3).

### 2.3. Identification of the BAHD Acyltransferase (BAHD) Gene Family

A total of 1741 *BAHD* family members were used in this analysis (Supplementary Figures S4 and S5). The numbers of retained *BAHD* members in *Arabidopsis thaliana* and *Populus trichocarpa* were similar to previous results [28,29], suggesting that our filtering stringency was conservative but appropriate. Some species included in this analysis had a lower number of *BAHD* family members than expected, possibly due to annotation issues.

**Figure 4:**
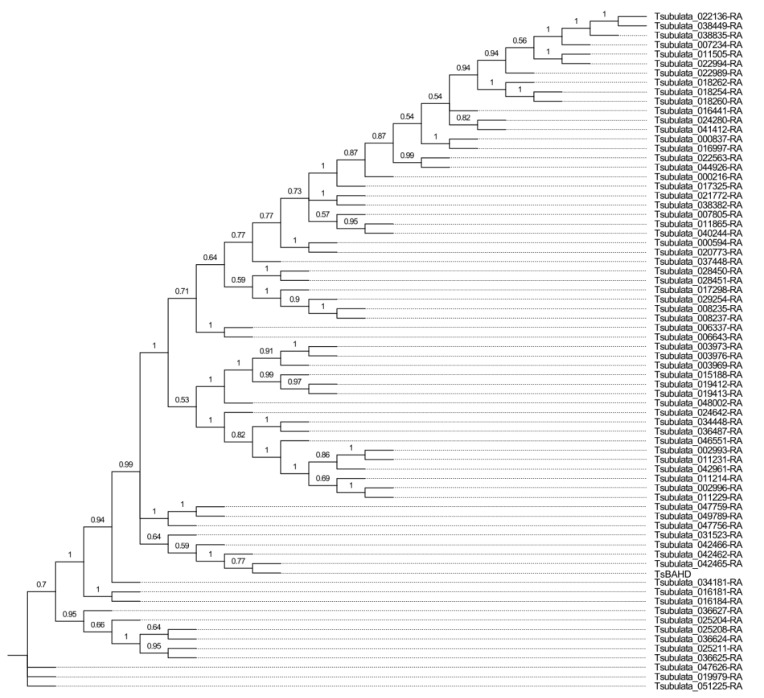
Phylogenetic relationship of the *BAHD* gene family in *Turnera subulata*. Generated using MEGA11 [30]. Values at nodes represent proportions from 1000 bootstrap replicates. Additional information related to motifs and intron/exon structure can be found in Supplementary Figure S6.

**Figure 5:**
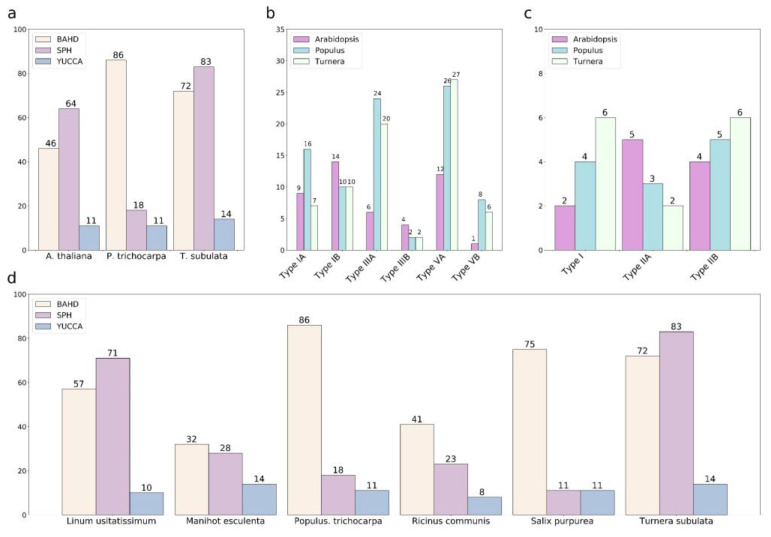
Total number of *BAHD*, *SPH*, and *YUCCA* family members in *Arabidopsis thaliana*, *Populus trichocarpa*, and *Turnera subulata* (**a**). Placement of the *BAHD* (**b**) and *YUCCA* (**c**) family members in their respective clades [29,31]. Clade IV of the *BAHD* family was not included as it is only present in monocots [29]. Clade II of the *BAHD* family could not be resolved. Total number of *BAHD*, *SPH,* and *YUCCA* family members in various species of Malpighiales (**d**).

We identified 72 putative *BAHD* family members in *Turnera* (Figure 4 and Supplementary Figure S6). The total number of *BAHD*s and assignment to the subclades were similar to *P. trichocarpa* (Figure 5a,b). *TsBAHD* falls into subclade IIIA.

### 2.4. Identification of the S-Protein Homolog (SPH) Gene Family

A total of 1552 *SPH* family members were used in this analysis (Supplementary Figures S4 and S7). Surprisingly, *SPH*s were identified in non-seed plants and “primitive” flowering plants *Amborella trichopoda* and *Nelumbo nucifera* but had apparently not been identified in monocots. Overall, the *SPH* tree had a large number of weakly supported branches comparable to the results of previous analyses and likely due to sequence divergence and/or the small size of the gene limiting phylogenetically informative sites [32,33].

**Figure 6:**
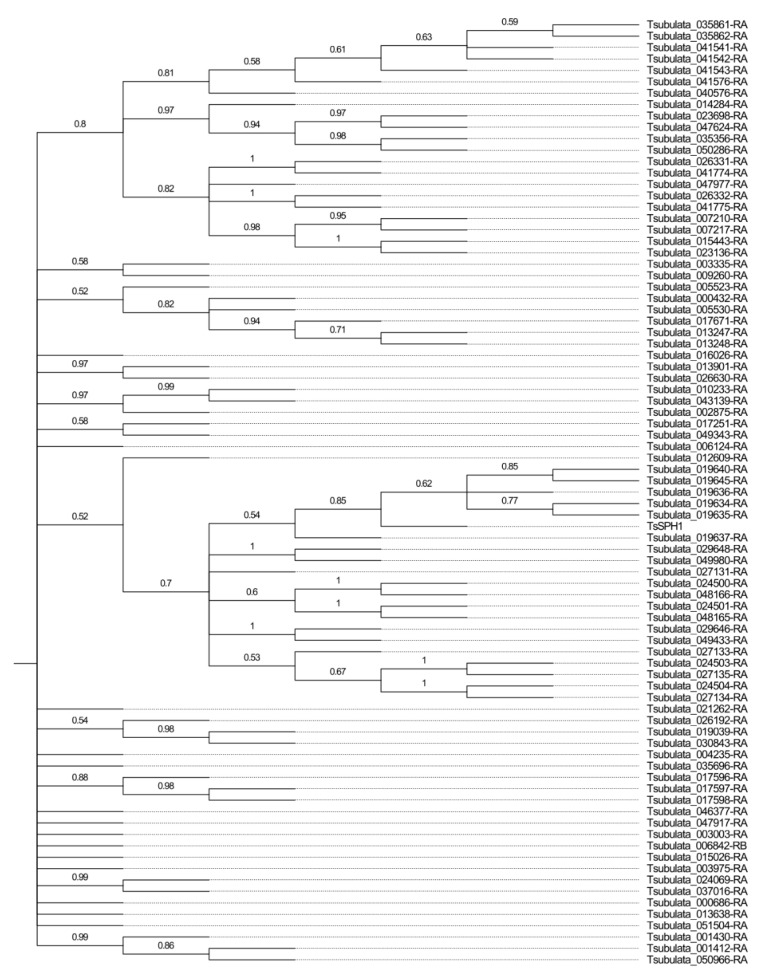
Phylogenetic relationships of the *SPH* gene family in *Turnera subulata*. Generated using MEGA11 [30]. Values at nodes represent proportions from 1000 bootstrap replicates. Additional information related to motifs and intron/exon structure can be found in Supplementary Figure S8.

We identified 83 putative *SPH* family members in *Turnera* (Figure 6 and Supplementary Figure S8). There have likely been several genome-specific duplication events, as is evident from the phylogenetic tree (Supplementary Figure S7). The number of *SPH* genes identified in *T. subulata* was similar to that of *Linum usitatissimum* (Figure 5c), which may suggest the expansion and/or contraction of the gene family in various lineages.

Interestingly, *TsSPH1′*s paralogs all reside on the same scaffold adjacent to one another (scaffold 850_149123). To determine if this region containing the SPHs evolved independently in *Turnera*, we used tBLASTn to identify *SPH* family members in *P. organensis*. We identified a region of a scaffold of *P. organensis*, scaffold4_size11498481 (GenBank JAEPBF010000262.1), that likely contains nine neighboring *SPH* family members. Once we had established the seven *T. subulata SPH*s that represented the family on scaffold 850_149123, we created a phylogeny using these genes from *T. subulata* and *P. organensis* (Supplementary Figure S9).

This analysis revealed that the region likely expanded in each species separately, as the *SPH*s form species-specific clades. Our results potentially suggest that the region, or the ancestral gene, is prone to tandem duplication events.

Previous studies [32] have suggested that monocots do not have *SPH* family members, despite the occurrence of *SPH* in some “primitive” angiosperms and non-seed plants (above). To determine if monocots truly lack the *SPH* gene family, we compared the *Arabidopsis thaliana SPH* family members to all monocot data in GenBank using tBLASTn searches, and also conducted some searches on CoGe. While we did not include *SPH* family members from any of the monocot genomes in our analyses above, we have now identified *SPH* family members in four monocot genomes, including *Elaeis guineensis* (Arecaceae), *Asparagus officinalis* (Asparagaceae), *Dioscorea cayenesis* (Dioscoreaceae), and *Eichhornia paniculata* (Pontederiaceae). The number of *SPH* genes for each species requires further investigation in these and other monocots. To determine if the homologs identified in *E. guineensis* (GenBank XM_010911761.2), *A. officinalis* (male genome GenBank XM_020420489.1 and female genome CoGe (ID35894)), *D. cayenesis* (GenBank XM_039285132.1), and *E. paniculata* (CoGe ID64302: Location: 5470690-5471133, Chromosome: PGA_scaffold6_pilon, Strand: 1) are truly *SPH* family members, we looked for the structural characteristics specific to *SPH* proteins/genes. All of these monocot *SPH*s appear to represent true *SPH* family members. *SPH*s may have been lost from several monocot lineages, but further investigation into the occurrence of this gene family is warranted.

### 2.5. Identification of the YUCCA (YUC) Gene Family

A total of 581 *YUCCA* family members were used in this analysis (Supplementary Figures S4 and S10). Previous analysis of the family in *A. thaliana* identified 11 family members [34], all of which were maintained after filtering. Previous analysis of the family in *P. trichocarpa* identified 12 family members [31], 11 of which were maintained after filtering.

**Figure 7:**
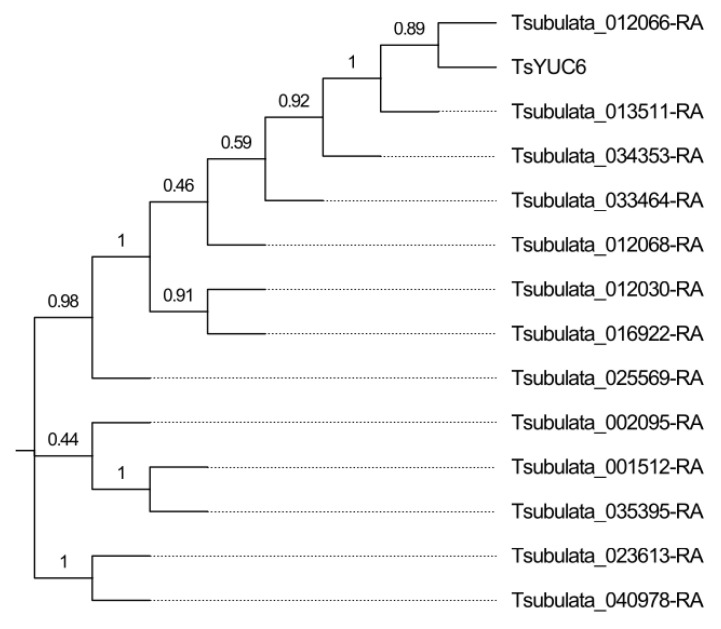
Phylogenetic relationship of the *YUCCA* gene family in *Turnera subulata*. Generated using MEGA11 [30]. Values at nodes represent proportions from 1000 bootstrap replicates. Additional information related to motifs and intron/exon structure can be found in Supplementary Figure S11.

This analysis identified 14 putative members of the *YUCCA* family in *Turnera subulata* (Figure 7 and Supplementary Figure S11). *TsYUC6* fell into the IIb clade. In *Arabidopsis,* the IIB clade is heavily involved in different aspects of floral development [34]. The closest *Arabidopsis* ortholog was *AtYUC6* (AT5G25620). There appears to be a *Turnera*-specific expansion of the family (Figure 5c).

In previous DEG analysis [16], Tsubulata_034353-RA, a homolog of *AtYUC2*, was identified as being depleted in the young stamen. This analysis strongly supports that *AtYUC2* (AT4G13260) is the closest homolog to Tsubulata_034353-RA. Tsubulata_034353-RA is of interest, as *AtYUC2* and *AtYUC6* are functionally redundant in *Arabidopsis* [34,35].

### 2.6. Possible Origin of the S-Genes

If the *S*-genes evolved after the splitting of Passifloroideae and Turneroideae, we would anticipate that the *S*-genes and their closest paralogs would share an ortholog in species of *Passiflora*. We searched for the *S*-genes’ closest paralogs in *P. organensis* and *P. edulis* (Supplementary Table S8). We also searched for the *S*-genes’ closest paralog in the two genomes. Our analysis revealed that all three *S*-genes and at least one of their paralogs shared a single ortholog in both *Passiflora* genomes. Our results suggest that the *S*-genes arose after the splitting of Turneroideae and Passifloroideae.

To determine if the *S*-genes arose sequentially, we located the *S*-genes’ closest paralogs in *T. subulata*. None of the *S*-genes’ closest paralogs reside on the same scaffold (Supplementary Table S4). Therefore, it is likely that the *S*-genes were duplicated independently in *T. subulata*. In an attempt to determine the order of acquisition, we calculated the synonymous substitution rates (K_s_) of the three *S*-genes and their closest paralogs (Supplementary Figure S12) following the methods of Gutiérrez-Valencia et al. [4]. *TsYUC6* in *T. subulata* has the greatest substitution rate (K_S_ = 0.628 ± 0.076), followed by *TsSPH1* (K_S_ = 0.589 ± −0.128) and finally *TsBAHD* (K_S_ = 0.511 ± 0.057). Limited data were available from the transcriptome of *T. joelii* where *TjYUC6* had K_S_ = 0.668 ± 0.08, comparable to that of *T. subulata*. We caution that this is an extremely preliminary analysis and would require further genome resources (e.g., well-assembled genome sequences for several distylous *Turnera* species) to mirror the more rigorous analysis carried out in *Primula* [11].

### 2.7. Expression Patterns of the BAHD and YUCCA Family

The expression patterns of *YUCCA* family members are generally organelle and tissue-specific [36,37], likely due to the number of critical roles that auxin plays. Previous analysis of the *YUCCA* family’s expression in *Arabidopsis,* maize, and rice suggests that ortholog expression differs between eudicots and monocots, but is similar between closely related species [38]. While the analysis by Matthes et al. [38] was not quantitative, such analyses can still provide meaningful insights into how expression patterns differ across species and aid in the formation of hypotheses regarding how gene families evolve within genomes [38]. Using similar methods, we wanted to determine if there were similarities in expression patterns of members of *BAHD* subclade IIIA and the *YUCCA* family in the floral tissues of *Populus* and *Turnera* compared to *Arabidopsis*. The *SPH* family was excluded, as the closest homologs of *TsSPH* are not expressed in floral tissues [16].

Several subgroups formed within *BAHD* subclade IIIA, each seeming to differ in expression pattern compared to other subgroups (Supplementary Figure S13). The group containing *TsBAHD* and *BIA1* (AT4G15400) showed a range of expression patterns within the floral tissues, with many of the genes showing higher expression patterns in the younger pistil.

Our analysis of the *YUCCA* family suggests that there has been conservation of expression, and likely function, of subclades I and IIB, but not IIA (Supplementary Figure S14). There was strong conservation in expression patterns of subclades IIB between all three species and strong conservation of subclade I between *Turnera* and *Populus.* Most of the *Turnera* and *Populus* genes assigned to subclade I are not expressed in the stamen or pistil. Subclade Class I of the *YUCCA* family is involved with shoot development and embryogenesis in *Arabidopsis* [39], while subclade IIB is involved in floral development [34].

In Class IIB, *Turnera* and *Populus* homologs of *AtYUC2* (AT5G25620) and *AtYUC6* (AT5G25620) showed a tendency towards higher expression in the stamen, while homologs of *AtYUC1* (AT4G32540) and *AtYUC4* (AT5G11320) showed a tendency towards higher expression in the pistil.

**Figure 8:**
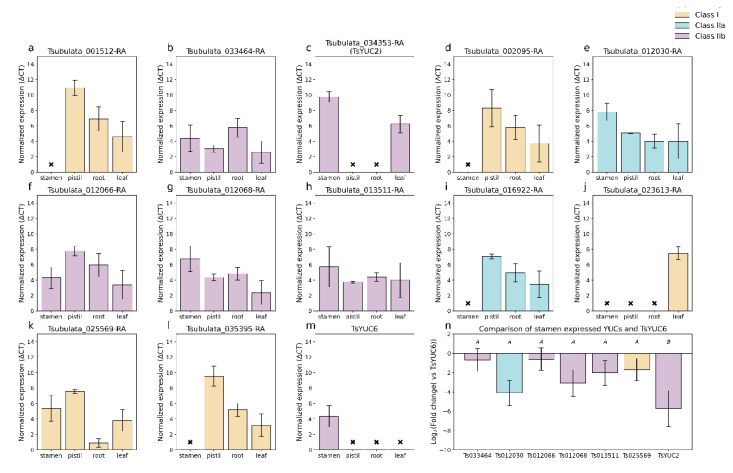
Expression of the *YUCCA* family in the developing leaves, apical root, immature stamen, and immature pistils of *Turnera subulata* (**a**–**m**). Bars are color-coded according to which *YUCCA* subfamily they belong to (see key). These values represent the average normalized expression, calculated using housekeeping genes *β-tubulin* and *UEVD1*. As these represent normalized expression (gene of interest—housekeeping gene), higher values represent lower levels of expression. X = not expressed. For (n), all means sharing a letter are not significantly different following a single factor ANOVA and Tukey’s test. F_7,16_ = 247.61, *p* < 0.0001. Values represent the Log_2_(Fold change). Error bars represent the standard error. Names were abbreviated in (n) to improve readability.

We used RT-qPCR to test hypotheses generated from previous analyses (Figure 8). For this analysis, we compared results with the tissue type that showed the highest expression level; our analysis was restricted to young leaves, apical roots, young pistils, and young stamens of the S-morph. Analysis was limited to the S-morph as the S-morph is hemizygous for the *S*-genes [3]. We opted to show averaged ΔCT values (i.e., normalized values) for Figure 8a–m, as we felt a comparison of expression of each gene across tissues did not accurately convey expression values. Note that ΔCT values are the inverse of expression levels (i.e., a high ΔCT value represents low expression).

Interestingly, many genes showed the highest expression in leaves (Figure 8a–j,l); this may suggest retention of the ancestral state as Class I and Class IIB in *Arabidopsis* are both involved in shoot development [39,40]. As we were particularly interested in *TsYUC6*, we compared expression values of the other stamen expressed genes with that of *TsYUC6* (Figure 8n). While Tsubulata_012066-RA shows peak expression in the leaves, its expression level in the stamen is approximately the same as *TsYUC6*. Tsubulata_013511-RA shows greater divergence in expression patterns, with the highest expression in the leaves and the lowest in the stamen.

Expression of *YUCCA* family member Tsubulata_40978-RA was not evaluated as specific primers could not be designed. RT-qPCR analysis was attempted for close paralogs of the *S*-genes *TsBAHD* and *TsSPH*, but we could not confidently amplify these genes specifically due to high levels of homology with other gene family members.

### 2.8. Identifying Hypothetical Neofunctionalization of the S-Genes

OrthoVenn2 identifies orthologous groups shared between species based on sequence homology [41]. Hypothetically, genes that group with one another should share a similar function. Once orthologous groups have been identified, the program identifies paralogous groups from the remaining genes. Any gene that does not fall into a paralogous group is denoted as a singleton and hypothetically has a unique function. As this analysis is used to determine hypothetical deviation in function from other homologs, and changes in expression are generally perceived as the first step in neofunctionalization [42], i.e., the evolution of a novel function, one may conclude that conservation in function suggests a more recent duplication event.

Of the three *S*-genes, *TsYUC6* and its closest paralog, Tsubulata_012066-RA, were both classified as singletons, while another close paralog, Tsubulata_013511-RA, falls into an orthologous group with the other five species (Supplementary Table S1). These results, in addition to our RT-qPCR and *YUCCA* gene phylogeny, may allude to complete neofunctionalization of Tsubulata_012066-RA and *TsYUC6*, i.e., in addition to altered expression, both may have developed an entirely new function. Furthermore, our results may suggest that Tsubulata_013511-RA has maintained the ancestral function of its *AtYUC6* ortholog, i.e., general male fertility.

*TsSPH1* formed a paralogous group with its five closest paralogs. Those *TsSPH1* paralogs all neighbor one another on the same scaffold; thus, they likely evolved via tandem duplication events. *Turnera* and *Passiflora SPH*s are in separate “orthologous” groups, and our OrthoVenn2 results suggest the potential neofunctionalization of the Turneroideae group, either by changes in expression pattern, signaling tag, or in function.

*TsBAHD* is part of an orthologous group with Tsubulata_042462-RA and homologs from the five species studied, though the *R. communis* homologs (29929.m004719 and 29929.m004743) are severely truncated and one *P. trichocarpa* member (POPTR_010G056400v3) is missing a *BAHD* family conserved motif [43], suggesting that these latter three genes may be nonfunctional. RNA-seq data showed that Tsubulata_042462-RA is also expressed in floral tissues, though it shows a tendency towards expression during early development and is expressed in both stamens and pistils. Tsubulata_042465-RA was classified as a singleton and is not expressed in floral tissues. The expression patterns of Tsubulata_042465-RA and the OrthoVenn2 results suggest that *TsBAHD* has undergone complete neofunctionalization (i.e., change in expression pattern in addition to change in general function). *TsBAHD*’s expression profile, its synonymous substitution rate, and OrthoVenn2 results suggest that the gene is more similar to Tsubulata_042462-RA and that its neofunctionalization likely relies solely on changes in gene expression.

**Figure 9:**
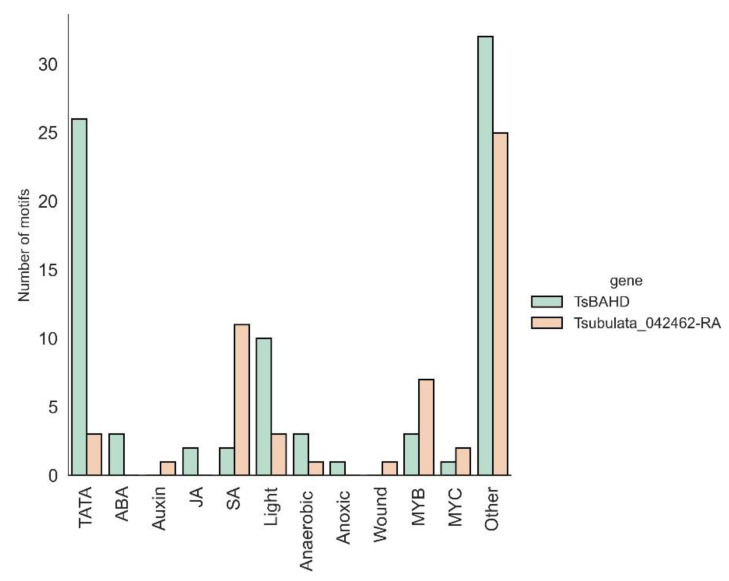
*Cis*-regulatory element motifs identified in the promoter region of *TsBAHD* and its closest paralog Tsubulata_042462-RA.

We used a series of bioinformatic tools to explore how the expression of *TsBAHD* may differ from Tsubulata_042462-RA. To predict subcellular localization based on *Arabidopsis* homology, we used WoLF PSORT [44]. Both *TsBAHD* and Tsubulata_042462-RA may localize to the nucleus or cytoplasm, though Tsubulata_042462-RA has a lower score and is apparently more promiscuous in terms of localization (Table 1). To investigate the potential effect of differences in cis-regulatory elements on the expression of *TsBAHD* and Tsubulata_042462-RA, we compared 1kb upstream regions of the two genes using PlantCARE [45]. We found tremendous differences between the upstream regions of the two genes (Figure 9, Supplementary Table S5).

## 3. Discussion

### 3.1. Repetitive Elements in the Turnera subulata Genome

Transposable elements (TE) are one of the driving forces of evolution, altering gene expression patterns [46,47,48], chromatin accessibility [45,49,50], and gene duplication and evolution [26,47]. It has been proposed that TEs may be the main driving force for genome evolution in several species, including *Moso bamboo* [51], *Pinus tabuliformis* [52], and *Allium fistulosum* [53]. The general importance of TE in the evolution of novel structures, such as fiber cells in cotton [46], flavor in onion [53], and self-compatibility in species of *Capsella* [54], may suggest they should be of interest in studies related to the evolution of the *S*-genes of distylous species. This is especially true considering that the *S*-loci of *Primula* [6], *Turnera* [3], and *Linum* [4] accumulate TEs.

*Passiflora* exhibits a wide range of genome size variation driven by the differential proliferation of Ty3/Gypsy (Ty3/gypsy-Tekay and Ty1/copia-Angela retrotransposons [26]. *T. subulata* has a genome size of 674.8 Mb, about twice the size of the smallest *Passiflora* genome (*P. organensis* 259 Mb) but considerably smaller than that of larger *P. quadrangularis* (2621 Mb) [26,55]. Interestingly, the repetitive sequence content of *T. subulata* is greater than other distylous species including *Primula veris* (46.07%) [11] and *Linum tenue* (49.36%) [4], a species with a comparable genome size of 702 Mb.

Class I repetitive elements were the most abundant (56.44%) in *T. subulata*, with COPIA elements making up 99.52 Mb or 17.73% of the genome. Similarly, 58.55% of repetitive elements fell into Class I in *P. organensis*; however, the majority fell into the subfamily GYPSY [25]. In both *Primula veris* and *Linum tenue,* Class I sequences were the most abundant, the majority of which were labeled as unclassified [4,11]. The large occurrence of GYPSY- and COPIA-type elements in *Turnera* and *Passiflora* is interesting, as these elements are potentially important for genome expansion [50]. Finally, a comparison of the *S*-locus haplotypes revealed a remarkable excess of TEs in the dominant vs. recessive haplotypes, consistent with expectations for TE element accumulation in non-recombining regions of genomes.

### 3.2. The S-Protein Homolog Gene Family Is Present in some Monocots

Previously, it was proposed by Rajaseker et al. that the *SPH* family may have been lost completely in monocots [33]. Our analysis of the *SPH* family initially identified *SPH* members only in non-seed plants, “primitive” angiosperms, and eudicots, but not monocots. It is possible that SPH homologs do occur in monocots but have diverged considerably in sequence and have gone unrecognized in annotations. It is documented that signaling peptides are under-annotated in model genomes, including *Arabidopsis thaliana* [56], *Zea mays* [57], and *Vitis vinifera* [58], and this is especially true for those with little sequence conservation [57]. As computational programs have difficulties annotating signaling peptides, and the SPH family has perhaps not been of particular interest outside of *Papaver* or *Turnera*, the family may be under-annotated in the species included in this analysis. This possibility is supported by the fact that in Arabidopsis, phylogenetic analysis has suggested that there are potentially 92 [33] to 100+ [59] *SPH* family members, but only 71 were annotated in the *A. thaliana* genome at the time of this analysis (Araport11 genome cDNA BLASTset 07-11-2019 release), suggesting that a significant number were missed in the process of annotation.

To determine if monocots have lost the *SPH* family, or if our analysis was biased by annotation issues in monocot genomes, we searched for family members across the monocot database on GenBank and CoGe. We identified four monocots, *Elaeis guineensis*, *Asparagus officinalis*, *Dioscorea cayenesis* (*rotundata*), and *Eichhornia paniculata*, that contain at least one *SPH* family member. These results suggest that the gene family may be present in other monocot families, and more detailed searches are required to verify the number of *SPH* genes in these and other monocot species. We found no evidence of *SPH* in the species of the Poales, suggesting that the gene family may have been lost from that lineage, though perhaps more rigorous searches are warranted.

### 3.3. The S-Locus Likely Evolved in Turneroideae via a Step-By-Step Manner Similar to the S-Loci from Primula and Linum

Kappel et al. [60] proposed two methods for how *S*-genes in distylous taxa are recruited to the pre-*S*-locus; sequentially via independent duplication events or via a segmental duplication event involving multiple (or all) *S*-genes. To determine if the *S*-locus arose by individual duplication events or a segmental duplication event, we identified the location of their closest paralogs in the *T. subulata* genome. The closest paralogs of all three *S*-genes reside on different scaffolds, suggesting that the *S*-genes must have arisen via three independent duplication events.

With the recent release of two *Passiflora* genomes [25,61], we were able to identify the location of the *S*-genes’ and their paralogs’ closest *Passiflora* orthologs. *TsBAHD* and its two closest homologs all shared a homolog in both species of *Passiflora* and *TsYUC6* and its closest homolog shared a homolog in both species of *Passiflora*.

As the *SPH* family likely underwent several *Turnera-*specific duplication events, we examined six paralogs of *SPH1*, all of which neighbor one another on a scaffold; interestingly, while *SPH1* shared the same *P. organensis* ortholog with three of its paralogs, it only shared a *P. edulis* ortholog with one of its paralogs, and this may be due to low conservation in sequence or perhaps gene loss. *P. organensis* apparently also has a region of its genome that contains the *Turnera* orthologs. Interestingly, phylogenetic analysis of these two regions suggests they evolved independently in *Passiflora* and *Turnera*, potentially suggesting that the region is prone to duplication events of *SPH*.

Our results suggest all three *S*-genes underwent duplication events after the divergence of Turneroideae and Passifloroideae, approximately 39.8–65.5 million years ago [62,63]. Additional genomic resources will be required to estimate the time of the duplication events.

To further test this hypothesis, we compared synonymous substitution rates between the three *S*-genes and their closest paralogs (for *SPH* and *BAHD*, the closest paralog was determined by calculating the synonymous substitution rate between the *S*-gene and all potential paralogs, the paralog with the smallest substitution rate was determined to be the closest paralog). We found varying rates across the three *S*-genes; *TsYUC6* showed the highest rate of synonymous substitution while *TsBAHD* showed the smallest. We also calculated synonymous substitution rates for a *Turnera joelii S*-gene and its respective paralog. Unfortunately, because only RNA-seq data from floral tissue are available, we could not perform this analysis for *TjBAHD* or *TjSPH1*, as the closest paralog to *TjBAHD* was artificially truncated and the closest paralogs to *TjSPH1* are not expressed in floral tissues. *TjYUC6* did show synonymous substitution rates similar to *TsYUC6* in *T. subulata*, supporting the analysis for the *YUC6* paralogs. We caution that this is an extremely preliminary analysis and additional genome resources would be required (e.g., well-assembled genomes for several distylous *Turnera* species) to mirror the more rigorous analysis carried out in *Primula* [11].

### 3.4. TsBAHD and TsSPH1 Likely Retained Their Ancestral Function, While TsYUC6 Likely Has Undergone Alteration of Function

The three *S*-genes likely arose via proximal, transposition, or dispersion duplication as they do not reside next to their closest paralogs; hence, it is likely that they have differences in, or entirely different, promoters than their closest paralogs, which would result in alterations of their expression patterns [42]. As such, deviation from their ancestral expression patterns is anticipated, but a complete change in function is not required for these genes to gain functionality in heterostyly.

Comparison of the *S*-genes’ expression patterns with their closest paralogs or orthologs may provide insight into their evolutionary history. Comparison of orthologous and paralogous groups may also provide insights into whether the *S*-genes may have hypothetically diverged from their ancestral function(s).

Our OrthoVenn2 results coupled with expression patterns suggest that *TsYUC6* and Tsubulata_012066-RA may have evolved new functions; however, it is likely that *TsYUC6* and Tsubulata_012066-RA, biochemically, are both still involved in the tryptophan-dependent auxin biosynthesis pathway. Knocking down *TjYUC6* in distylous *Turnera joelii* alters the expression of auxin-related genes [15], as expected for a gene involved in auxin metabolism. In addition, to our knowledge, all *YUCCA* family members characterized to date are enzymatically involved in the tryptophan-dependent auxin biosynthesis pathway. Hence, the results presented here suggest that additional research is warranted to fully characterize the enzymatic and biochemical properties of *TsYUC6* and Tsubulata_012066-RA.

*TsSPH1* and its paralogs formed a paralogous group, suggesting a shared function of the group that differs from the other analyzed species. It is not surprising that the paralogs may share a function, as they likely evolved via tandem duplication events based on their genomic locations [42]; this is not the case for *TsSPH1*, as it resides on a different genomic scaffold. *TsSPH1*’s new genomic location may have altered its expression pattern, i.e., filament expression, as close paralogs are not expressed in the stamen or pistils of *T. subulata* or *T. joelii* [16]. This suggests that its role in distyly is likely a result of alteration in expression profile rather than in biochemical function.

*TsBAHD* formed an orthologous group composed of proteins from all of the analyzed species. This suggests a much smaller degree of divergence than observed in the other *S*-genes, supporting the hypothetical order of origin of the *S*-genes. Our results suggest that the origin of *TsBAHD* is similar to that of *TsSPH1*, i.e., *TsBAHD* has retained its ancestral biochemical function and the alteration of expression pattern is responsible for neofunctionalization of this gene. This is supported by the heterologous expression of *TsBAHD* and *TkBAHD*, an ortholog from distylous *Turnera krapovickasii*. Heterologous expression of these genes in *Arabidopsis thaliana* resulted in a dwarf phenotype and alteration in the expression of BR-responsive genes [13,14], similar to its *Arabidopsis* homolog *BIA1* [64].

These findings suggest that the ancestral role of *AtBIA1*, *TsBAHD*, and close paralogs was to regulate or respond to BR levels, perhaps in floral tissues. Two close paralogs, AT5G47980 [65,66] and AT3G26040 [67], are likely regulated by cytokinin (CK). Changes in expression in response to CK are of interest as there is considerable cross-talk between BR and CK, typically involving the two phytohormones working in conjunction [68]. Additionally, one member of this group, AT5G23970, is a flower-specific gene [69].

As *TsBAHD* and Tsubulata_042462-RA are both expressed in the pistil throughout development, neofunctionalization of *TsBAHD* may rely heavily on subcellular-localized expression or differences in the promoter. While both enzymes are anticipated to localize to the nucleus and cytosol, though Tsubulata_042462-RA to a lower extent, we did identify several differences in the 1 kb upstream regions of the two genes. The abundance of salicylic acid (SA)-related motifs in Tsubulata_042462-RA may suggest that its expression is heavily regulated by SA, potentially suggesting that Tsubulata_042462-RA is involved in stress tolerance or pathogen defense, as SA is known to regulate genes related to stress and pathogen response [70]. As other members of the *BAHD* family are involved in plant immunity [71], it would not be farfetched to hypothesize that Tsubulata_042462-RA is involved in pathogen defense. If this is the case, it would suggest that *BAHD*’s SI response/self-recognition may have evolved from a pathogen recognition system, though this is highly speculative. Interestingly, many light-responsive motifs were identified in *BAHD*’s promoter region. This is of particular interest, as it has been proposed that PIF signaling hubs, which initiate growth in response to light, are repressed in the style of the S-morph, potentially due to inactivation of BR by *BAHD* [16].

## 4. Materials and Methods

### 4.1. Genome Assembly

gDNA of *T. subulata* (plant F60SS;3) was isolated from nuclei to minimize organellar DNA contamination [72] as follows: Young leaves were ground directly in liquid nitrogen. Ground plant material was resuspended in nuclei isolation buffer and filtered with a Miracloth (Millipore). Triton X-100 was added to lyse any organelles. Samples were then centrifuged to pellet nuclei. Finally, gDNA was extracted using the previously described CTAB extraction protocol [73].

Three libraries were constructed: a PCR-free Illumina shotgun library (average insert size of ~450 bp) sequenced with Illumina HiSeq Rapid PE250 and two Nextera mate pair libraries (insert size ~5 kb), the first of which was sequenced with Illumina HiSeq Rapid PE250, and the second using Illumina HiSeq 2500 PE125 (Supplementary Table S6).

Default parameters were used for all software, including those used in annotation, unless otherwise noted. De novo assembly of reads from the PCR-free Illumina shotgun library was carried out using DISCOVAR de novo (v.2013) [74]. The resulting contigs were then scaffolded using the mate pair reads which were first filtered with NextCLIP [75]. Scaffolding was performed using SSPACE [76] (minimum links = 3, maximum link ratio = 0.85), and gaps were then filled using GapCloser (v.1.12) [77] (max read length = 155 bp). An additional round of scaffolding was finally performed.

### 4.2. Genome Annotation

Only scaffolds 10 kb or greater in size were annotated, as smaller ones rarely contained genes. We used portions of the pipelines of both Funannotate (v1.7.4) [78] and MAKER (v.2.31.8) [79] for genome annotation (Supplementary Figure S15) as described below.

A *Turnera subulata*-specific repeat library was generated using RepeatModeler (v.1.0.9) [80]. This library was combined with a library of plant-specific repeat elements [81] before being used as input to RepeatMasker (v.4.0.7) [82] for scaffold masking.

The Funannotate pipeline was used to generate a genome-guided RNA-seq assembly (PASA v2.4.1; Trinity v2.8.5) [83,84] using the RNA-seq reads from all the previously published *T. subulata* S-morph samples (PRJNA589060). A total of 320,484,056 trimmed read pairs were normalized (yielding 13,442,338 read pairs) and used in a genome-guided transcriptome assembly.

Next, we trained two in silico gene predictors, Snap and AUGUSTUS. Snap (v. 2006-07-28) [85] was trained using PASA gene models in Funannotate. BRAKER (v.2.1.4) [86] was used to train AUGUSTUS (v3.3.3) parameters [87] using eight iterations to optimize AUGUSTUS parameters.

MAKER (v.2.31.8) [88] was used for gene prediction with the option to use tRNAscan-SE [89] for tRNA gene annotation. In addition to the earlier generated items (see above and Supplementary Figure S15), we provided a protein database composed of all protein sequences from the *Arabidopsis thaliana* TAIR10 collection (v.2019-97-11) [90], *Populus trichocarpa* (UP000006729) and *Vitis vinifera* (UP000009183). These represent proteins from three well-annotated genomes with both *P. trichocarpa* and *Vitis vinifera* from the same order (Malpighiales) as *Turnera*.

We examined genes/proteins predicted by MAKER [89] and used InterProScan (v.5.40-77.0) [91] to identify Pfam domains in the predicted proteins and SignalP to identify signaling peptides (v.4.1) [92]. We filtered the proteins, inspecting both their Annotation Edit Distance scores (AED) and whether they possessed a Pfam domain. AED estimates the quality of prediction [88]; an AED score of 0 indicates a perfect fit, while 1 indicates a lack of fit. Genes with AED scores of 1.0 were removed, unless they possessed a Pfam domain, following the recommendation in Campbell et al. 2014 (basic protocol 5).

Despite our efforts to repeat-mask the genome, some predicted proteins/genes possessed Pfam domains, indicating they were likely TEs. We removed those proteins/genes from the final annotation. The remaining set of genes/proteins and tRNAs were then formatted for input to Funannotate, for final preparation of the annotation.

We used the annotate command of Funannotate, and input the filtered genes/proteins and tRNAs from MAKER, InterProScan results, and annotation information from EggNOG (v5.0) [93]. We ran the command line program tbl2asn (NCBI) to flag any errors in the annotation format prior to genome submission. Genes flagged were further edited as necessary, using BLAST and FGENESH+ [94] to infer intron/exon splice sites.

We used the PiRATE pipeline [95] to detect, classify, and enumerate various kinds of transposable elements in the genome.

### 4.3. Putative BAHD, SPH, and YUCCA Identification in Turnera subulata

Predicted *BAHD* acyltransferase, *Self-incompatibility Protein Homolog* (*SPH*), and *YUCCA* family members were pulled from the annotated *T. subulata* genome (PRJNA626617). To confirm that the putative *BAHD* and *YUCCA* are part of their respective predicted families, FIMO (version 5.3.3) of the MEME suite [96] was used with default settings to identify the previously described BAHD [43] and YUCCA family [97] motifs. Proteins whose predicted motifs had a *p*-value of < 0.05 were considered for further analysis.

As the conserved region of the *SPH* family is ambiguous [33], potential members of the *SPH* family were confirmed using sequence homology with *A. thaliana* genes labeled as *SPH* (Araport11 genome cDNA blastset 07-11-2019 release). For further confirmation, peptide sequences were examined for typical structural features of the SPH family [33]. Secondary structures of the putative SPH members were predicted using NetSurfP-2.0 on default settings [98] to identify β-strands.

### 4.4. Data retrieval, Alignment, and Phylogenetic Analysis

All sequences annotated as members of the *BAHD*, *SPH*, or *YUCCA* families were downloaded from Phytozome (V. 12.1.6) [99]; this included 56 plant species ranging from nonvascular plants to eudicots (Supplementary Figure S4). Sequences were aligned using Muscle (version 3.8.31) [100]. Problematic sequences (those that appeared to be partial, containing potential but ambiguous introns, did not align well, or were missing the family motifs) were removed from further analyses. As the aspartic acid residue of the DFGWG motif is predicted to be involved in proper formation of the solvent channel of BAHD [101], any protein whose DFGWG domain had non-conserved amino acid substitutions was removed from further analysis.

Maximum likelihood gene family trees were generated using RAxML [102] on the CIPRES Science Gateway server. A total of 100 bootstrap replicates were used for all trees. *T. subulata*-specific gene trees were generated using MEGA X [103]. The peptide sequences for each gene were first aligned using MUSCLE. A maximum likelihood tree was then generated using the bootstrap method (1000 replicates); all other settings used were left on default. 

To identify potential duplication events prior to the divergence of *Turnera* and *Passiflora*, CoGE:Blast [104] was used to identify the location of the *S*-genes’ closest homologs in the recently published *Passiflora edulis* [60] and *P. organensis* [25] genomes.

OrthoVenn2 was used to identify orthologous clusters shared between P. edulis [60], *P. organensis* [25], Populus trichocarpa, Manihot esculenta, and Ricinus communis. Both Passiflora datasets were downloaded from CoGE:Blast; *R. communis* [21] was downloaded from Phytozome. *P. trichocarpa* and *M. esculenta* datasets are currently available in OrthoVenn2. Identification of orthologous clusters provides information on the quality of annotation and can aid in the identification of GO terms important for species of interest.

Synonymous substitution rates were determined using methods outlined in Gutiérrez-Valencia et al. 2022 with slight modifications. Codons were aligned using Clustal and substitution rates were calculated using the Nei–Gojobori method with 1000 bootstrap replicates using MEGA11 [30]. Standard errors are based on the 1000 replicates.

### 4.5. Expression Comparison across Turnera, Populus, and Arabidopsis

Expression data from *T. subulata* (PRJNA589060) [16], *A. thaliana* [105], and *P. trichocarpa* were downloaded from Phytozome. Expression ratios were determined by summing expression data for a given gene and assigning percentage values.

### 4.6. RT-qPCR Analysis of Putative YUCCA Family Members

*Turnera subulata* (4x) was grown in greenhouse conditions (Pullman, WA, USA). Three biological replicates of stamen and pistils from immature buds (3 mm), immature leaves (10 mm), and root apex were collected. RNA isolation, cDNA synthesis, and RT-qPCR were performed as previously described [16]. RT-qPCR results were normalized using the housekeeping genes *UEV1D* and *β-Tubulin*. Primers and RT-qPCR conditions can be found in Supplementary Tables S7 and S8.

## 5. Conclusions

Here, we present the annotation of the draft genome of *Turnera subulata*, a valuable resource for future comparative studies. Additionally, we present the first phylogenetic analysis of the *BAHD, SPH,* and *YUCCA* gene families in *T. subulata*. Our analyses support and refine the placement of the *S*-genes in their respective gene families, clarifying paralog relationships. These results support the function of *TsBAHD* as an acyltransferase involved in BR degradation [13,14], similar to its *Arabidopsis* homolog *BIA1* [63]; *TsSPH1* as a cysteine-rich signaling peptide; and *TsYUC6* as a flavin-containing monooxygenase, similar to its *Arabidopsis* homolog *YUC6* [106].

Our analyses suggest that the three *S*-genes were acquired independently via three separate duplication events after the divergence of Turneroideae and Passifloroideae, as the *S*-genes and their respective paralog(s) share a single homolog in two *Passiflora* genomes. Identification of the *S*-gene paralogs suggests that the *S*-locus evolved in a step-by-step manner, similar to that of *Primula* and *Linum*. Analysis of orthologs and expression patterns suggests that *TsYUC6* is the only *S*-gene to have possibly evolved a new function; the neofunctionalization of *TsSPH1* and *TsBAHD* likely relies solely on alterations in expression patterns. Further sequencing and chromosome-level assembly of other related genomes will be necessary to characterize the origin of the *S*-locus with greater confidence in *Turnera*.

## Figures and Tables

**Table 1:** WoLF PSORT predicted subcellular location of *TsBAHD* and its paralog.

Gene	Predicted Subcellular Location
*TsBAHD*	nucl: 8, cyto: 5
Tsubulata_042462-RA	nucl: 6, nucl_plas: 5, cyto: 2, mito: 2, plas: 2, chlo: 1

Numbers refer to the number of features found in the respective gene associated with a given subcellular location.

## Data Availability

The annotated genome of *Turnera subulata* has been uploaded to NCBI’s GenBank (PRJNA626617).

## Supplementary Materials

The following supporting information can be downloaded at: https://www.mdpi.com/article/10.3390/plants12020286/s1, Table S1: OrthoVenn2 generated clusters containing *S*-genes; Table S2: Updated nomenclature for previously identified differentially expressed genes; Table S3: CoGE:BLAST identified paralogs in *Passiflora organensis* and *Passiflora edulis*; Table S4: Members of the *BAHD*, *SPH*, and *YUCCA* families residing on the same scaffold; Table S5: Cis-regulatory Element Motifs in the 1 kb upstream region of *TsBAHD* and Tsubulata_042462-RA; Table S6: Information regarding library construction; Table S7: ABI conditions for RT-qPCR; Table S8: Primers used in RT-qPCR; Figure S1: Synteny of the region flanking the S-locus with closely related species; Figure S2: Distribution of synonymous substation rates of genes flanking the *S*-locus; Figure S3: OrthoVenn2 results of the Turneroideae and Passifloroideae comparison; Figure S4: Common tree of species used across phylogenetic analyses; Figure S5: Phylogenetic tree of the BAHD family across species downloaded from Phytozome; Figure S6: Phylogenetic relationship and motif annotations of putative *BAHD* family members in *Turnera subulata*; Figure S7: Phylogenetic tree of the SPH family across species downloaded from Phytozome; Figure S8: Phylogenetic relationship and predicted secondary structure annotations of putative *SPH* family members in *Turnera subulata*; Figure S9: Phylogeny of the *SPH*s in *Passiflora* and *Turnera*; Figure S10: Phylogenetic tree of the YUCCA family across species downloaded from Phytozome; Figure S11: Phylogenetic relationship and motif annotations of putative *YUCCA* family members in *Turnera subulata*; Figure S12: Synonymous substitution rates (Ks) for the three *S*-genes relative to their closest paralogs; Figure S13: Comparative analysis of the expression of the IIIA subclade of the *BAHD* family across *Arabidopsis thaliana*, *Populus trichocarpa*, and *Turnera subulata*; Figure S14: Comparative analysis of the expression of the *Yucca* family across *Arabidopsis thaliana*, *Populus trichocarpa*, and *Turnera subulata*; Figure S15: Pipeline used for genome annotation. References [107, 108] are references in the supplemental material.

## References

[1] Barrett S.C.H. (2019). ‘A Most Complex Marriage Arrangement’: Recent Advances on Heterostyly and Unresolved Questions. New Phytol..

[2] Li J., Cocker J.M., Wright J., Webster M.A., McMullan M., Dyer S., Swarbreck D., Caccamo M., van Oosterhout C., Gilmartin P.M. (2016). Genetic Architecture and Evolution of the *S* Locus Supergene in *Primula vulgaris*. Nat. Plants.

[3] Shore J.S., Hamam H.J., Chafe P.D.J., Labonne J.D.J., Henning P.M., McCubbin A.G. (2019). The Long and Short of the *S*-locus in *Turnera* (Passifloraceae). New Phytol..

[4] Gutiérrez-Valencia J., Fracassetti M., Berdan E.L., Bunikis I., Soler L., Dainat J., Kutschera V.E., Losvik A., Désamoré A., Hughes P.W. (2022). Genomic Analyses of the *Linum* Distyly Supergene Reveal Convergent Evolution at the Molecular Level. Curr. Biol..

[5] Yasui Y., Mori M., Aii J., Abe T., Matsumoto D., Sato S., Hayashi Y., Ohnishi O., Ota T. (2012). *S-LOCUS EARLY FLOWERING 3* Is Exclusively Present in the Genomes of Short-Styled Buckwheat Plants That Exhibit Heteromorphic Self-Incompatibility. PLoS ONE.

[6] Nowak M.D., Russo G., Schlapbach R., Huu C.N., Lenhard M., Conti E. (2015). The Draft Genome of *Primula veris* Yields Insights into the Molecular Basis of Heterostyly. Genome Biol..

[7] Burrows B.A., McCubbin A.G. (2017). Sequencing the Genomic Regions Flanking *S*-Linked *PvGLO* Sequences Confirms the Presence of Two *GLO* Loci, One of Which Lies Adjacent to the Style-Length Determinant Gene *CYP734A50*. Plant Reprod..

[8] Huu C.N., Keller B., Conti E., Kappel C., Lenhard M. (2020). Supergene Evolution via Stepwise Duplications and Neofunctionalization of a Floral-Organ Identity Gene. Proc. Natl. Acad. Sci. USA.

[9] Huu C.N., Kappel C., Keller B., Sicard A., Takebayashi Y., Breuninger H., Nowak M.D., Bäurle I., Himmelbach A., Burkart M. (2016). Presence versus Absence of *CYP734A50* Underlies the Style-Length Dimorphism in Primroses. eLife.

[10] Huu C.N., Plaschil S., Himmelbach A., Kappel C., Lenhard M. (2021). Female Self-Incompatibility Type in Heterostylous *Primula* Is Determined by the Brassinosteroid-Inactivating Cytochrome P450 *CYP734A50*. Curr. Biol..

[11] Potente G., Léveillé-Bourret É., Yousefi N., Choudhury R.R., Keller B., Diop S.I., Duijsings D., Pirovano W., Lenhard M., Szövényi P. (2022). Comparative Genomics Elucidates the Origin of a Supergene Controlling Floral Heteromorphism. Mol. Biol. Evol..

[12] Ushijima K., Nakano R., Bando M., Shigezane Y., Ikeda K., Namba Y., Kume S., Kitabata T., Mori H., Kubo Y. (2012). Isolation of the Floral Morph-related Genes in Heterostylous Flax (*Linum grandiflorum*): The Genetic Polymorphism and the Transcriptional and Post-transcriptional Regulations of the *S* Locus. Plant J..

[13] Matzke C.M., Shore J.S., Neff M.M., McCubbin A.G. (2020). The *Turnera* Style *S*-Locus Gene *TsBAHD* Possesses Brassinosteroid-Inactivating Activity When Expressed in *Arabidopsis thaliana*. Plants.

[14] Matzke C.M., Hamam H.J., Henning P.M., Dougherty K., Shore J.S., Neff M.M., McCubbin A.G. (2021). Pistil Mating Type and Morphology Are Mediated by the Brassinosteroid Inactivating Activity of the *S*-Locus Gene *BAHD* in Heterostylous *Turnera* Species. Int. J. Mol. Sci..

[15] Henning P.M., Shore J.S., McCubbin A.G. (2022). The *S*-Gene *YUC6* Pleiotropically Determines Male Mating Type and Pollen Size in Heterostylous *Turnera* (Passifloraceae): A Novel Neofunctionalization of the *YUCCA* Gene Family. Plants.

[16] Henning P.M., Shore J.S., McCubbin A.G. (2020). Transcriptome and Network Analyses of Heterostyly in *Turnera subulata* Provide Mechanistic Insights: Are *S*-Loci a Red-Light for Pistil Elongation?. Plants.

[17] Stern D.L. (2013). The Genetic Causes of Convergent Evolution. Nat. Rev. Genet..

[18] Hao Y., Qu Y., Song G., Lei F. (2019). Genomic Insights into the Adaptive Convergent Evolution. Curr. Genom..

[19] Sackton T.B., Clark N. (2019). Convergent Evolution in the Genomics Era: New Insights and Directions. Philos. Trans. R. Soc. B Biol. Sci..

[20] Manni M., Berkeley M.R., Seppey M., Simão F.A., Zdobnov E.M. (2021). BUSCO Update: Novel and Streamlined Workflows along with Broader and Deeper Phylogenetic Coverage for Scoring of Eukaryotic, Prokaryotic, and Viral Genomes. Mol. Biol. Evol..

[21] Chan A.P., Crabtree J., Zhao Q., Lorenzi H., Orvis J., Puiu D., Melake-Berhan A., Jones K.M., Redman J., Chen G. (2010). Draft Genome Sequence of the Oilseed Species *Ricinus communis*. Nat. Biotechnol..

[22] Motamayor J.C., Mockaitis K., Schmutz J., Haiminen N., Iii D.L., Cornejo O., Findley S.D., Zheng P., Utro F., Royaert S. (2013). The Genome Sequence of the Most Widely Cultivated *Cacao* Type and Its Use to Identify Candidate Genes Regulating Pod Color. Genome Biol..

[23] International Cassave Genetic Map Consortium (2014). High-resolution linage map and chromosome-scale genome assembly for cassava (*Manihot esculenta* Crantz) from 10 populations. G3 Genes Genomes Genet..

[24] Tuskan G.A., DiFazio S., Jansson S., Bohlmann J., Grigoriev I., Hellsten U., Putnam N., Ralph S., Rombauts S., Salamov A. (2006). The Genome of Black Cottonwood, *Populus trichocarpa* (Torr. & Gray). Science.

[25] Costa Z.P., Varani A.M., Cauz-Santos L.A., Sader M.A., Giopatto H.A., Zirpoli B., Callot C., Cauet S., Marande W., Souza Cardoso J.L. (2021). A Genome Sequence Resource for the Genus *Passiflora*, the Genome of the Wild Diploid Species *Passiflora organensis*. Plant Genome.

[26] Sader M., Vaio M., Cauz-Santos L.A., Dornelas M.C., Vieira M.L.C., Melo N., Pedrosa-Harand A. (2021). Large vs Small Genomes in *Passiflora*: The Influence of the Mobilome and the Satellitome. Planta.

[27] Ou S., Su W., Liao Y., Chougule K., Agda J.R.A., Hellinga A.J., Lugo C.S.B., Elliott T.A., Ware D., Peterson T. (2019). Benchmarking Transposable Element Annotation Methods for Creation of a Streamlined, Comprehensive Pipeline. Genome Biol..

[28] Yu X.-H., Gou J.-Y., Liu C.-J. (2009). BAHD Superfamily of Acyl-CoA Dependent Acyltransferases in *Populus* and *Arabidopsis*: Bioinformatics and Gene Expression. Plant Mol. Biol..

[29] Tuominen L.K., Johnson V.E., Tsai C.-J. (2011). Differential Phylogenetic Expansions in BAHD Acyltransferases across Five Angiosperm Taxa and Evidence of Divergent Expression among *Populus* Paralogues. BMC Genom..

[30] Tamura K., Stecher G., Kumar S. (2021). MEGA11: Molecular Evolutionary Genetics Analysis Version 11. Mol. Biol. Evol..

[31] Ye X., Kang B., Osburn L.D., Li Y., Zong-Ming (Max) C. (2009). Identification of the Flavin-Dependent Monooxygenase-Encoding *YUCCA* Gene Family in *Populus trichocarpa* and Their Expression in Vegetative Tissues and in Response to Hormone and Environmental Stresses. Plant Cell Tissue Organ Cult. PCTOC.

[32] Kakeda K., Jordan N.D., Conner A., Ride J.P., Franklin-Tong V.E., Franklin F.C.H. (1998). Identification of Residues in a Hydrophilic Loop of the *Papaver rhoeas* S Protein That Play a Crucial Role in Recognition of Incompatible Pollen. Plant Cell.

[33] Rajasekar K.V., Ji S., Coulthard R.J., Ride J.P., Reynolds G.L., Winn P.J., Wheeler M.J., Hyde E.I., Smith L.J. (2019). Structure of SPH (Self-Incompatibility Protein Homologue) Proteins: A Widespread Family of Small, Highly Stable, Secreted Proteins. Biochem. J..

[34] Cheng Y., Dai X., Zhao Y. (2006). Auxin Biosynthesis by the YUCCA Flavin Monooxygenases Controls the Formation of Floral Organs and Vascular Tissues in *Arabidopsis*. Genes Dev..

[35] Cecchetti V., Celebrin D., Napoli N., Ghelli R., Brunetti P., Costantino P., Cardarelli M. (2017). An Auxin Maximum in the Middle Layer Controls Stamen Development and Pollen Maturation in *Arabidopsis*. New Phytol..

[36] Zhao Y. (2018). Essential Roles of Local Auxin Biosynthesis in Plant Development and in Adaptation to Environmental Changes. Annu. Rev. Plant Biol..

[37] Blakeslee J.J., Rossi T.S., Kriechbaumer V. (2019). Auxin Biosynthesis: Spatial Regulation and Adaptation to Stress. J. Exp. Bot..

[38] Matthes M.S., Best N.B., Robil J.M., Malcomber S., Gallavotti A., McSteen P. (2019). Auxin EvoDevo: Conservation and Diversification of Genes Regulating Auxin Biosynthesis, Transport, and Signaling. Mol. Plant.

[39] Cheng Y., Dai X., Zhao Y. (2007). Auxin Synthesized by the YUCCA Flavin Monooxygenases Is Essential for Embryogenesis and Leaf Formation in *Arabidopsis*. Plant Cell.

[40] Chen Q., Dai X., De-Paoli H., Cheng Y., Takebayashi Y., Kasahara H., Kamiya Y., Zhao Y. (2014). Auxin Overproduction in Shoots Cannot Rescue Auxin Deficiencies in Arabidopsis Roots. Plant Cell Physiol..

[41] Xu L., Dong Z., Fang L., Luo Y., Wei Z., Guo H., Zhang G., Gu Y.Q., Coleman-Derr D., Xia Q. (2019). OrthoVenn2: A Web Server for Whole-Genome Comparison and Annotation of Orthologous Clusters across Multiple Species. Nucleic Acids Res..

[42] Wang Y., Wang X., Paterson A.H. (2012). Genome and Gene Duplications and Gene Expression Divergence: A View from Plants: Gene Duplication and Expression Divergence. Ann. N. Y. Acad. Sci..

[43] D’Auria J.C. (2006). Acyltransferases in Plants: A Good Time to Be BAHD. Curr. Opin. Plant Biol..

[44] Horton P., Park K.-J., Obayashi T., Fujita N., Harada H., Adams-Collier C.J., Nakai K. (2007). WoLF PSORT: Protein Localization Predictor. Nucleic Acids Res..

[45] Lescot M. (2002). PlantCARE, a database of plant cis-acting regulatory elements and a portal to tools for in silico analysis of promoter sequences. Nucleic Acids Res..

[46] Lanciano S., Cristofari G. (2020). Measuring and Interpreting Transposable Element Expression. Nat. Rev. Genet..

[47] Wang K., Huang G., Zhu Y. (2016). Transposable Elements Play an Important Role during Cotton Genome Evolution and Fiber Cell Development. Sci. China Life Sci..

[48] Zhao D., Ferguson A.A., Jiang N. (2016). What Makes up Plant Genomes: The Vanishing Line between Transposable Elements and Genes. Biochim. Biophys. Acta BBA Gene Regul. Mech..

[49] Zhang A., Zhang W. (2022). Characterization of Transposon-Derived Accessible Chromatin Regions in Rice (*Oryza sativa*). Int. J. Mol. Sci..

[50] Noshay J.M., Marand A.P., Anderson S.N., Zhou P., Mejia Guerra M.K., Lu Z., O’Connor C.H., Crisp P.A., Hirsch C.N., Schmitz R.J. (2021). Assessing the Regulatory Potential of Transposable Elements Using Chromatin Accessibility Profiles of Maize Transposons. Genetics.

[51] Ramakrishnan M., Yrjälä K., Satheesh V., Zhou M.-B., Cho J. (2021). Bamboo Transposon Research: Current Status and Perspectives. Plant Transposable Elements.

[52] Niu S., Li J., Bo W., Yang W., Zuccolo A., Giacomello S., Chen X., Han F., Yang J., Song Y. (2022). The Chinese Pine Genome and Methylome Unveil Key Features of Conifer Evolution. Cell.

[53] Liao N., Hu Z., Miao J., Hu X., Lyu X., Fang H., Zhou Y.-M., Mahmoud A., Deng G., Meng Y.-Q. (2022). Chromosome-Level Genome Assembly of Bunching Onion Illuminates Genome Evolution and Flavor Formation in *Allium* Crops. Nat. Commun..

[54] Ågren J., Wang W., Koenig D., Neuffer B., Weigel D., Wright S.I. (2014). Mating System Shifts and Transposable Element Evolution in the Plant Genus *Capsella*. BMC Genom..

[55] López A., Panseri A.F., Poggio L., Fernández A. (2011). Nuclear DNA Content in the Polyploid Complex *Turnera ulmifolia* (*Turnera* L., Passifloraceae). Plant Syst. Evol..

[56] Lease K.A., Walker J.C. (2006). The Arabidopsis Unannotated Secreted Peptide Database, a Resource for Plant Peptidomics. Plant Physiol..

[57] Wang S., Tian L., Liu H., Li X., Zhang J., Chen X., Jia X., Zheng X., Wu S., Chen Y. (2020). Large-Scale Discovery of Non-Conventional Peptides in Maize and Arabidopsis through an Integrated Peptidogenomic Pipeline. Mol. Plant.

[58] Pei M.-S., Liu H.-N., Wei T.-L., Yu Y.-H., Guo D.-L. (2022). Large-Scale Discovery of Non-Conventional Peptides in Grape (*Vitis vinifera* L.) through Peptidogenomics. Hortic. Res..

[59] Ride J.P., Davies E.M., Franklin F.C.H., Marshall D.F. (1999). Analysis of Arabidopsis Genome Sequence Reveals a Large New Gene Family in Plants. Plant Mol. Biol..

[60] Kappel C., Huu C.N., Lenhard M. (2017). A Short Story Gets Longer: Recent Insights into the Molecular Basis of Heterostyly. J. Exp. Bot..

[61] Xia Z., Huang D., Zhang S., Wang W., Ma F., Wu B., Xu Y., Xu B., Chen D., Zou M. (2021). Chromosome-Scale Genome Assembly Provides Insights into the Evolution and Flavor Synthesis of Passion Fruit (*Passiflora edulis* Sims). Hortic. Res..

[62] Muschner V.C., Zamberlan P.M., Bonatto S.L., Freitas L.B. (2012). Phylogeny, Biogeography and Divergence Times in *Passiflora* (Passifloraceae). Genet. Mol. Biol..

[63] Thulin M., Razafimandimbison S.G., Chafe P., Heidari N., Kool A., Shore J.S. (2012). Phylogeny of the Turneraceae Clade (Passifloraceae s.l.): Trans-Atlantic Disjunctions and Two New Genera in Africa. TAXON.

[64] Wang M., Liu X., Wang R., Li W., Rodermel S., Yu F. (2012). Overexpression of a Putative Arabidopsis BAHD Acyltransferase Causes Dwarfism That Can Be Rescued by Brassinosteroid. J. Exp. Bot..

[65] Kiba T., Naitou T., Koizumi N., Yamashino T., Sakakibara H., Mizuno T. (2005). Combinatorial Microarray Analysis Revealing Arabidopsis Genes Implicated in Cytokinin Responses through the His→Asp Phosphorelay Circuitry. Plant Cell Physiol..

[66] Lee D.J., Park J.-Y., Ku S.-J., Ha Y.-M., Kim S., Kim M.D., Oh M.-H., Kim J. (2007). Genome-Wide Expression Profiling of *ARABIDOPSIS RESPONSE REGULATOR 7(ARR7*) Overexpression in Cytokinin Response. Mol. Genet. Genom..

[67] Černý M., Kuklová A., Hoehenwarter W., Fragner L., Novák O., Rotková G., Jedelský P.L., Žáková K., Šmehilová M., Strnad M. (2013). Proteome and Metabolome Profiling of Cytokinin Action in Arabidopsis Identifying Both Distinct and Similar Responses to Cytokinin Down- and up-Regulation. J. Exp. Bot..

[68] Saini S., Sharma I., Pati P.K. (2015). Versatile Roles of Brassinosteroid in Plants in the Context of Its Homoeostasis, Signaling and Crosstalks. Front. Plant Sci..

[69] Shi J.X., Malitsky S., De Oliveira S., Branigan C., Franke R.B., Schreiber L., Aharoni A. (2011). SHINE Transcription Factors Act Redundantly to Pattern the Archetypal Surface of Arabidopsis Flower Organs. PLoS Genet..

[70] Janda T., Szalai G., Pál M. (2020). Salicylic Acid Signalling in Plants. Int. J. Mol. Sci..

[71] Zheng Z., Qualley A., Fan B., Dudareva N., Chen Z. (2009). An Important Role of a BAHD Acyl Transferase-like Protein in Plant Innate Immunity. Plant J..

[72] Williamson R.J., Josephs E.B., Platts A.E., Hazzouri K.M., Haudry A., Blanchette M., Wright S.I. (2014). Evidence for Widespread Positive and Negative Selection in Coding and Conserved Noncoding Regions of *Capsella grandiflora*. PLoS Genet..

[73] Doyle J.J., Doyle J.L. (1987). A Rapid DNA Isolation Procedure for Small Quantities of Fresh Leaf Tissue. Phytochem. Bull..

[74] Weisenfeld N.I., Yin S., Sharpe T., Lau B., Hegarty R., Holmes L., Sogoloff B., Tabbaa D., Williams L., Russ C. (2014). Comprehensive Variation Discovery in Single Human Genomes. Nat. Genet..

[75] Leggett R.M., Clavijo B.J., Clissold L., Clark M.D., Caccamo M. (2014). NextClip: An Analysis and Read Preparation Tool for Nextera Long Mate Pair Libraries. Bioinformatics.

[76] Boetzer M., Henkel C.V., Jansen H.J., Butler D., Pirovano W. (2011). Scaffolding Pre-Assembled Contigs Using SSPACE. Bioinformatics.

[77] Goodman L., Edmunds S.C., Basford A.T. (2012). Large and Linked in Scientific Publishing. GigaScience.

[78] Palmer J.M., Stajich J. (2012). Nextgenusfs/Funannotate: Funannotate v1.7.4 2020. Goodman L, Edmunds SC, Basford AT. Large and linked in scientific publishing. Gigascience.

[79] Holt C., Yandell M. (2011). MAKER2: An Annotation Pipeline and Genome-Database Management Tool for Second-Generation Genome Projects. BMC Bioinform..

[80] Smit A.F.A., Hubley R. RepeatModeler Open-1.0 2008–2015. http://www.repeatmasker.org.

[81] Merchant N., Lyons E., Goff S., Vaughn M., Ware D., Micklos D., Antin P. (2016). The IPlant Collaborative: Cyberinfrastructure for Enabling Data to Discovery for the Life Sciences. PLoS Biol..

[82] Smit A.F.A., Hubley R., Green P. RepeatMasker Open-4.0 2013–2015. http://www.repeatmasker.org.

[83] Haas B.J. (2003). Improving the Arabidopsis Genome Annotation Using Maximal Transcript Alignment Assemblies. Nucleic Acids Res..

[84] Grabherr M.G., Haas B.J., Yassour M., Levin J.Z., Thompson D.A., Amit I., Adiconis X., Fan L., Raychowdhury R., Zeng Q. (2011). Full-Length Transcriptome Assembly from RNA-Seq Data without a Reference Genome. Nat. Biotechnol..

[85] Korf I. (2004). Gene Finding in Novel Genomes. BMC Bioinform..

[86] Hoff K.J., Lange S., Lomsadze A., Borodovsky M., Stanke M. (2016). BRAKER1: Unsupervised RNA-Seq-Based Genome Annotation with GeneMark-ET and AUGUSTUS: Table 1. Bioinformatics.

[87] Stanke M., Keller O., Gunduz I., Hayes A., Waack S., Morgenstern B. (2006). AUGUSTUS: Ab Initio Prediction of Alternative Transcripts. Nucleic Acids Res..

[88] Campbell M.S., Holt C., Moore B., Yandell M. (2014). Genome Annotation and Curation Using MAKER and MAKER-P. Curr. Protoc. Bioinforma..

[89] Chan P.P., Lowe T.M., Kollmar M. (2019). TRNAscan-SE: Searching for TRNA Genes in Genomic Sequences. Gene Prediction.

[90] Berardini T.Z., Reiser L., Li D., Mezheritsky Y., Muller R., Strait E., Huala E. (2015). The Arabidopsis Information Resource: Making and Mining the “Gold Standard” Annotated Reference Plant Genome: Tair: Making and Mining the “Gold Standard” Plant Genome. Genesis.

[91] Quevillon E., Silventoinen V., Pillai S., Harte N., Mulder N., Apweiler R., Lopez R. (2005). InterProScan: Protein Domains Identifier. Nucleic Acids Res..

[92] Nielsen H., Kihara D. (2017). Predicting Secretory Proteins with SignalP. Protein Function Prediction.

[93] Huerta-Cepas J., Szklarczyk D., Heller D., Hernández-Plaza A., Forslund S.K., Cook H., Mende D.R., Letunic I., Rattei T., Jensen L.J. (2019). EggNOG 5.0: A Hierarchical, Functionally and Phylogenetically Annotated Orthology Resource Based on 5090 Organisms and 2502 Viruses. Nucleic Acids Res..

[94] Solovyev V., Kosarev P., Seledsov I., Vorobyev D. (2006). Automatic Annotation of Eukaryotic Genes, Pseudogenes and Promoters. Genome Biol..

[95] Berthelier J., Casse N., Daccord N., Jamilloux V., Saint-Jean B., Carrier G. (2018). A transposable element annotation pipeline and expression analysis reveal potentially active elements in the microalga *Tisochrysis lutea*. Genomics..

[96] Grant C.E., Bailey T.L., Noble W.S. (2011). FIMO: Scanning for Occurrences of a given Motif. Bioinformatics.

[97] Expósito-Rodríguez M., Borges A.A., Borges-Pérez A., Pérez J.A. (2011). Gene Structure and Spatiotemporal Expression Profile of Tomato Genes Encoding YUCCA-like Flavin Monooxygenases: The ToFZY Gene Family. Plant Physiol. Biochem..

[98] Klausen M.S., Jespersen M.C., Nielsen H., Jensen K.K., Jurtz V.I., Sønderby C.K., Sommer M.O.A., Winther O., Nielsen M., Petersen B. (2019). NetSurfP-2.0: Improved Prediction of Protein Structural Features by Integrated Deep Learning. Proteins Struct. Funct. Bioinforma..

[99] Goodstein D.M., Shu S., Howson R., Neupane R., Hayes R.D., Fazo J., Mitros T., Dirks W., Hellsten U., Putnam N. (2012). Phytozome: A Comparative Platform for Green Plant Genomics. Nucleic Acids Res..

[100] Edgar R.C. (2004). MUSCLE: Multiple Sequence Alignment with High Accuracy and High Throughput. Nucleic Acids Res..

[101] Morales-Quintana L., Moya-León M.A., Herrera R. (2015). Computational Study Enlightens the Structural Role of the Alcohol Acyltransferase DFGWG Motif. J. Mol. Model..

[102] Stamatakis A. (2014). RAxML Version 8: A Tool for Phylogenetic Analysis and Post-Analysis of Large Phylogenies. Bioinformatics.

[103] Kumar S., Stecher G., Li M., Knyaz C., Tamura K. (2018). MEGA X: Molecular Evolutionary Genetics Analysis across Computing Platforms. Mol. Biol. Evol..

[104] Grover J.W., Bomhoff M., Davey S., Gregory B.D., Mosher R.A., Lyons E. (2017). CoGe LoadExp+: A Web-based Suite That Integrates Next-generation Sequencing Data Analysis Workflows and Visualization. Plant Direct.

[105] Waese J., Fan J., Pasha A., Yu H., Fucile G., Shi R., Cumming M., Kelley L.A., Sternberg M.J., Krishnakumar V. (2017). EPlant: Visualizing and Exploring Multiple Levels of Data for Hypothesis Generation in Plant Biology. Plant Cell.

[106] Cecchetti V., Altamura M.M., Falasca G., Costantino P., Cardarelli M. (2008). Auxin Regulates *Arabidopsis* Anther Dehiscence, Pollen Maturation, and Filament Elongation. Plant Cell.

[107] Veltri D., Wight M.M., Crouch J.A. (2016). SimpleSynteny: A Web-Based Tool for Visualization of Microsynteny across Multiple Species. Nucleic Acids Res..

[108] Hu B., Jin J., Guo A.-Y., Zhang H., Luo J., Gao G. (2015). GSDS 2.0: An Upgraded Gene Feature Visualization Server. Bioinformatics.

