# Supplementary material for: Annotation of the Turnera subulata (Passifloraceae) Draft Genome Reveals the S-Locus Evolved after the Divergence of Turneroideae from Passifloroideae in a Stepwise Manner
Source: Plants (Basel). 2023 Jan 7;12(2):286. doi: 10.3390/plants12020286 (PMC9862265; doi:10.3390/plants12020286)
Supplement: Supplementary file 1 [file plants-12-00286-s001.zip › plants-2098363-supplementary (2)/Supplementary.pdf]

Supplementary Table S1. OrthoVenn2 generated clusters containing *S*-genes

S-gene	Other proteins	Associated enriched GO term
BAHD	Ricinus: 29929.m004719<br>Ricinus: 29929.m004743<br>Manihot: MANES_12G016900<br>Populus: POPTR_010G056300v3<br>Populus: POPTR_010G056400v3<br>Passiflora: CDS_3194772138_12614<br>Passiflora: CDS_3194753076_23485<br>Turnera: Tsubulata_042462-RA	GO:0009820; P:alkaloid metabolic process
SPH1	Turnera: Tsubulata_19640-RA<br>Turnera: Tsubulata_19645-RA<br>Turnera: Tsubulata_19636-RA<br>Turnera: Tsubulata_19634-RA<br>Turnera: Tsubulata_19635-RA<br>Turnera: Tsubulata_19637-RA	GO:0060320; P:rejection of self-pollen

Supplementary Table S2. Updated nomenclature for previously identified differentially expressed genes [16].

2020 Name	Current Name	Family	Young Stamen	Young pistil	Mature stamen	Mature pistil	Arabidopsis homolog
TsBAHD	*TsBAHD*	BAHD	-	Enriched	-	Enriched	AT5G47980
Tsub_00000135-RA	Tsubulata_000216-RA	BAHD	Enriched	-	-	-	AT5G17540
Tsub_00011564-RA	Tsubulata_019412-RA	BAHD	-	-	-	Enriched	AT3G29590
Tsub_00021993-RA	Tsubulata_036487-RA	BAHD	-	-	-	Depleted	AT5G23940
Tsub_00022067-RA	Tsubulata_036624-RA	BAHD	-	Depleted	-	-	AT3G26040
Tsub_00024268-RA	Tsubulata_040244-RA	BAHD	-	-	-	Depleted	AT2G25150
Tsub_00027864-RA	Tsubulata_046551-RA	BAHD	Depleted	-	-	-	AT3G50280
TsSPH1	*TsSPH1*	SPH	Enriched	-	Enriched	-	AT4G16295
Tsub_00002485-RA	Tsubulata_004235-RA	SPH	-	-	-	Depleted	AT5G38700
Tsub_00003537-RA	Tsubulata_006124-RA	SPH	Enriched	-	Enriched	-	AT4G16295
Tsub_00011356-RA	Tsubulata_019039-RA	SPH	Enriched	-	-	-	AT4G16295
Tsub_00020676-RA	Tsubulata_034353-RA	YUCCA	Depleted	-	-	-	AT4G13260
TsYUC6	*TsYUC6*	YUCCA	Enriched	-	Enriched	-	AT5G25620

Supplementary Table S3. CoGE:BLAST [105] identified paralogs in *Passiflora organensis* and *Passiflora edulis*.

	T. subulata	***P. organensis***			***P. edulis***		
		**Scaffold**	**Position**	**E-value**	**Scaffold**	**Position**	**E-value**
**BAHD**	TsBAHD	scaffold194_size94437	17202	0	GWHAZTM00000001	179143548	0
	Tsubulata_042462-RA	scaffold194_size94437	17202	2E-172	GWHAZTM00000001	179143548	2E-175
	Tsubulata_042465-RA	scaffold194_size94437	17202	0	GWHAZTM00000001	179143548	0
**SPH**	TsSPH1	scaffold4_size11498481	11471255	4E-49	GWHANWG00000004	81883317	3E-46
	Tsubulata_019634-RA	scaffold4_size11498481	11454556	6E-43	GWHANWG00000004	81883287	6E-43
	Tsubulata_019635-RA	scaffold22_size2164848	1019431	7E-43	GWHANWG00000004	81883323	7E-44
	Tsubulata_019636-RA	scaffold4_size11498481	11454592	6E-45	GWHANWG00000004	81883317	2E-45
	Tsubulata_019637-RA	scaffold4_size11498481	11454556	3E-16	GWHANWG00000004	81883287	1E-15
	Tsubulata_019640-RA	scaffold4_size11498481	11471123	3E-31	GWHANWG00000004	81883188	3E-30
	Tsubulata_019645-RA	scaffold4_size11498481	11471123	3E-30	GWHANWG00000004	78962794	4E-30
**YUC**	TsYUC6	scaffold8_size8616334	3241333	3E-138	GWHAZTM00000001	198171934	5E-124
	Tsubulata_012066-RA	scaffold8_size8616334	3241333	1E-144	GWHAZTM00000001	198171979	1E-130
	Tsubulata_013511-RA	scaffold8_size8616334	3241333	5E-153	GWHAZTM00000009	4433551	7E-142

Supplementary Table S4. Members of the *BAHD*, *SPH*, and *YUCCA* families residing on the same scaffold.

	BAHD	***SPH***	***YUCCA***
scf62_sz374423	Tsubulata_002993-RA<br>Tsubulata_002996-RA	Tsubulata_003003-RA	-
scf91_sz346335	Tsubulata_003969-RA<br>Tsubulata_003973-RA<br>Tsubulata_003976-RA	Tsubulata_003975-RA	-
scf5190_sz28544	Tsubulata_047626-RA	Tsubulata_047624-RA	-
scf1941_sz87093	-	*TsSPH1*[*]	*TsYUC6*[*]

* *S*-genes

Supplementary Table S5. *Cis*-regulatory Element Motifs in the 1kb upstream region of *TsBAHD* and Tsubulata_042462-RA

***Cis*-element**	**Description (where applicable)**	**TsBAHD**	**Tsubulata_042462-RA**
ABRE	ABA responsive element	3	0
AE-Box	Light responsive element	1	0
ARE	anaerobic induction	3	1
AT~TATA-box		1	3
AuxRR-core	Auxin response	0	1
Box 4	Light responsive element	4	0
CAAT-box	Common *cis*-element in promoter and enhancer regions	12	16
CCAAT-box	MYBHv1 binding site	0	1
CGTCA-motif	MeJA-responsiveness	1	0
ERE		1	0
G-Box	Light responsive element	3	0
GC-motif	Anoxic specific induction	1	0
MBS	MYB binding site involved in drought-inducibility	1	0
GT1-motif	Light responsive element	0	1
I-Box	Light responsive element	0	1
MYB		0	3
MYB recognition site		0	1
MYB like sequence		0	2
MYC		1	2
Myb		2	0
O2-site	Zein metabolism regulation	1	2
STRE		3	2
Sp1	Light responsive element	1	0
TATA		2	0
TATA-box		23	0
TCA-element	Salicylic acid response	2	11
TCT-motif	Light response	1	1
TGACG-motif	MeJA response	1	0
Unnamed_2	*Zea mays* identified element	0	1
Unnamed__4	*Petroselinum hortense* identified element	13	3
WRE3		0	1
WUN-Motif	Wound-responsive element	0	1
as-1		1	0
dOCT		1	0

Supplementary Table S6. Information regarding library construction.

	**Library 1**	**Library 2**	**Library 3**
Method	HiSeq Rapid PE250	Illumina HiSeq 2500 PE125	HiSeq Rapid PE125
Total paired-end reads	15734190	279876652	72685390
Read average quality		34	
Library	Nexetera mate pair		PCR-free Illumina shotgun
Library average insert size	c. 5kb		c. 450bp

Supplementary Table S7. ABI Conditions for RT-qPCR

**Stage**	**Temperature**	**Time**
Holding	95.0 °C	10:00
Cycling (40 cycles)	95.0 °C	00:15
	61.5 °C	00:30
Melt Curve		
Step 1	95.0 °C 100%	00:15
Step 2	61.5 °C 1%	01:00
Step 3	95.0 °C 100%	00:15
Step 4	61.5 °C	00:15

Supplementary Table S8. Primers used in RT-qPCR

**Transcript**	**Forward (5' → 3')**	**Reverse (5' → 3')**
Tsubulata_002095-RA	GAAGTGCTCATTGTCGGTG	AGGGCATGTAAGGAAGCTG
Tsubulata_016922-RA	ACATGTTTCAACACTCAGGTCA	CAAGGATAACGAATGGGACA
Tsubulata_033464-RA	GTTCCCTTCTTGATCCTTGAG	AGGAGGGAATGGCATGT
Tsubulata_023613-RA	AGAAAGAAATCCAGGTACTTCCA	TGCTTAGGCAATCCACTAACA
Tsubulata_035395-RA	CCACCATTGGAGAGTCAGA	CGTTAAACTAATGTCGGTTGCT
Tsubulata_012066-RA	TGCCAAAAGAGTAGCCGA	ACCTCTTCGAATATCGCATG
Tsubulata_013511-RA	TGGACTGCTTGAGAGAAATAGAA	AGAGGCCACGCAGCT
Tsubulata_001512-RA	GAAGCACTGTGAATTATTGGCT	TTCATTCTTACTCGCAGCTAACT
Tsubulata_025569-RA	ACGGGGAAGACCTACCAG	AGCAGTTTTAGACCAATGTAAACC
Tsubulata_012068-RA	CAAGGCCAATGATTTTGC	TCCATTGATCTGCCACATC
Tsubulata_012030-RA	CTCTCCCAAATTTGGCAAG	ACCGTTAACCCAAATGCAC
Tsubulata_034353-RA	TCGCTACGGGCTACAAAAGCAC	CGTTAAACTAATGTCGGTTGCT
TsYUC6	GATGGATTCCCTAGAGGCAC	CACCTTCCGATATCTTCTGCT
β-tubulin	AGATTTTCCGACCCGACA	GCAATCACAATCTCGGCT
UEVD1	AGCCAGCCAAGTCGTCT	CATCGTCCATTCCATAGCTTAC

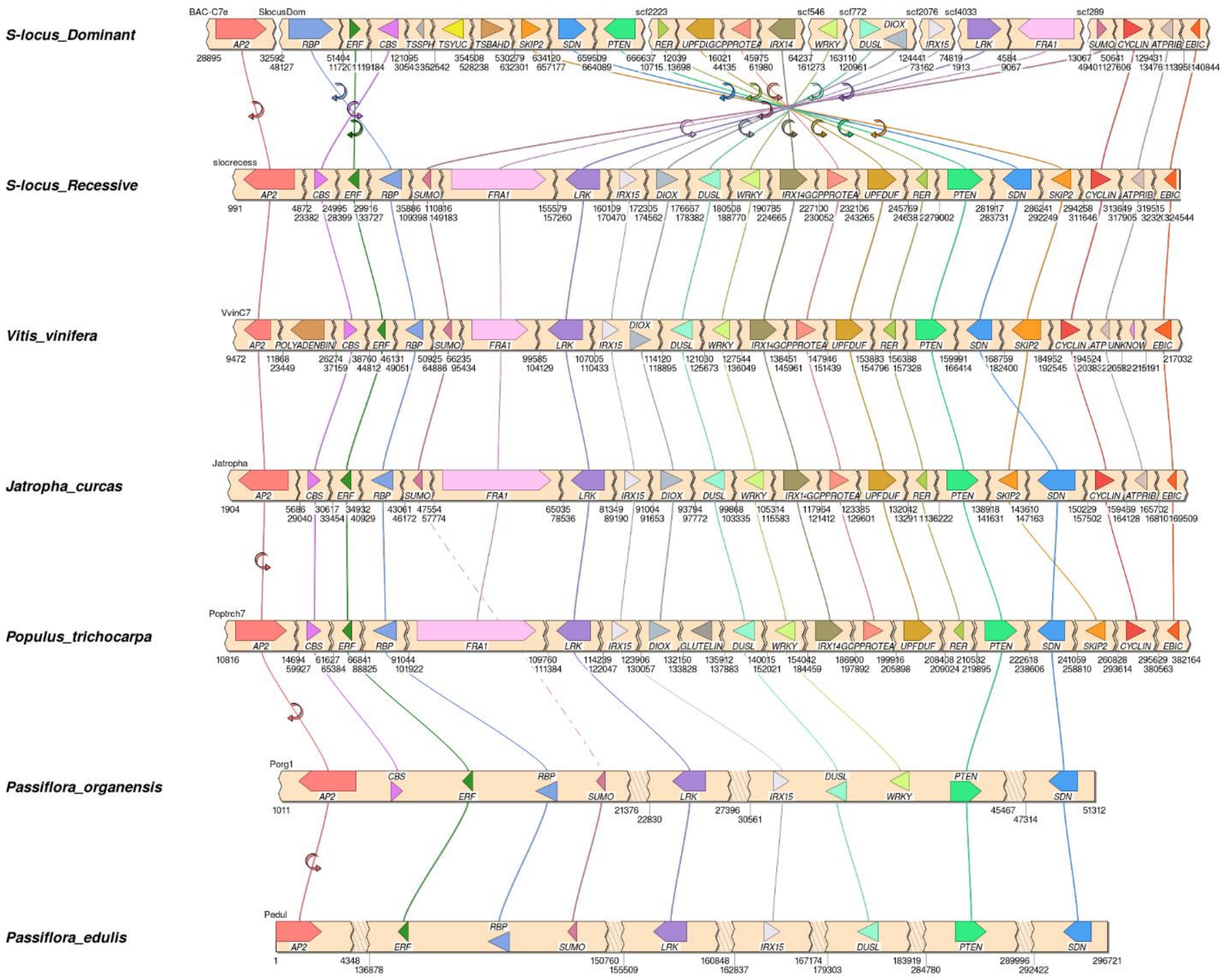

Supplemental Figure S1. Synteny of the region flanking the *S*-locus with closely related species. We used SimpleSynteny [108] to compare the *S*-locus haplotypes of *Turnera subulata* to scaffolds from the genomes of five species of Malpighiales including *Vitis vinifera*, *Jatropha curcas*, *Populus trichocarpa*, *Passiflora organensis* and *Passiflora edulis*. The recessive *S*-locus of *Turnera subulata* shows remarkable collinearity with scaffolds of *Vitis vinifera*, *Jatropha curcas*, and *Populus trichocarpa* while dominant *S*-locus haplotype possess a number of rearrangements as previously reported [3]. The dominant haplotype is comprised of 8 scaffolds, and the orientation and order of some scaffolds isn't known with certainty. Interestingly, scaffolds from both *Passiflora organensis* and *Passiflora edulis* appear to have lost a number of genes from this region, relative to the other Malpighiales.

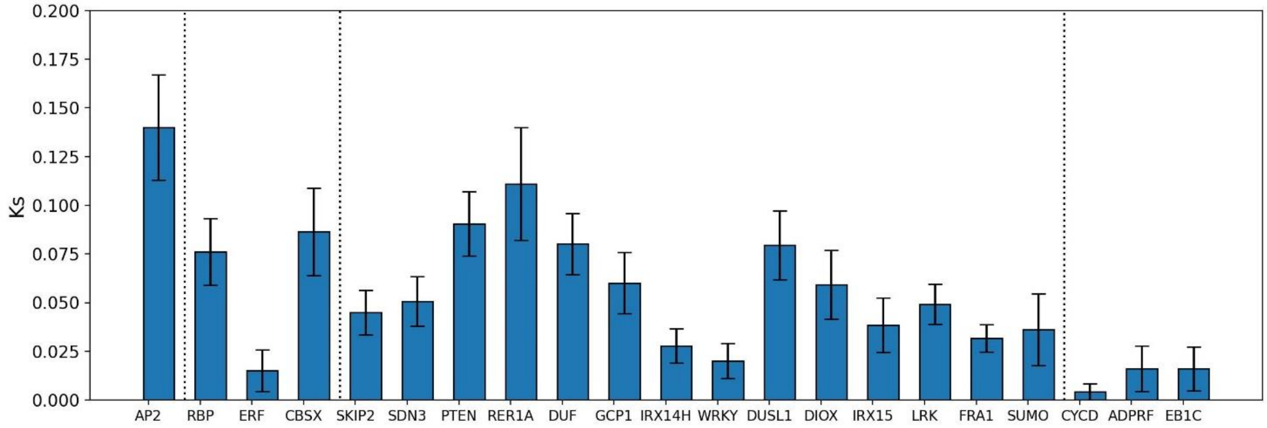

Supplemental Figure S2. Distribution of synonymous substation rates of genes flanking the *S*-locus. Distribution of synonymous substitution rates, Ks, for genes on the *S*-locus haplotypes other than the 3 hemizygous genes. Dashed lines represent the points of inversion. Substitution rates were estimated as described in the methods for the comparison of paralogs.

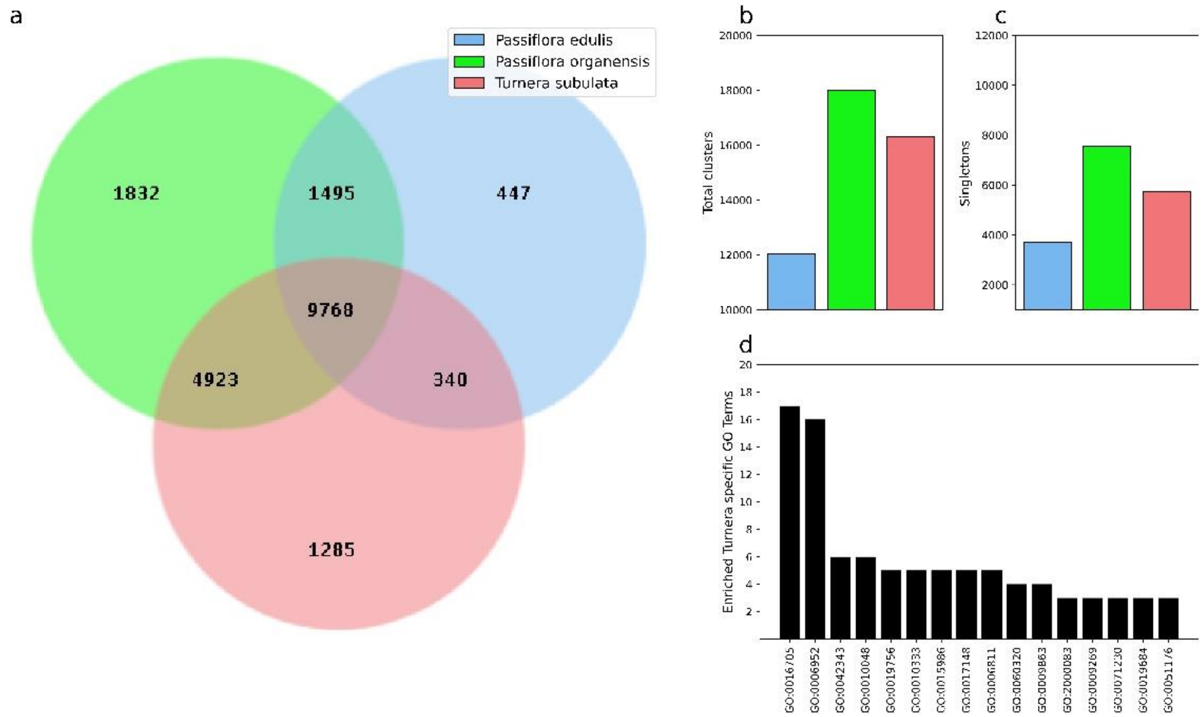

S**upplementary Figure S3.** OrthoVenn2 results of the Turneroideae and Passifloroideae comparison. Venn diagrams showing orthologous groups shared between the three species or proteins that were specific to one species (a). Total number of clusters for each species including both orthologous and paralogous clusters (b). Total singletons for each species, these represent proteins that are predicted to not share a similar function with other orthologs or paralogs (c). Enriched GO terms for *Turnera* specific paralogous groups (d).

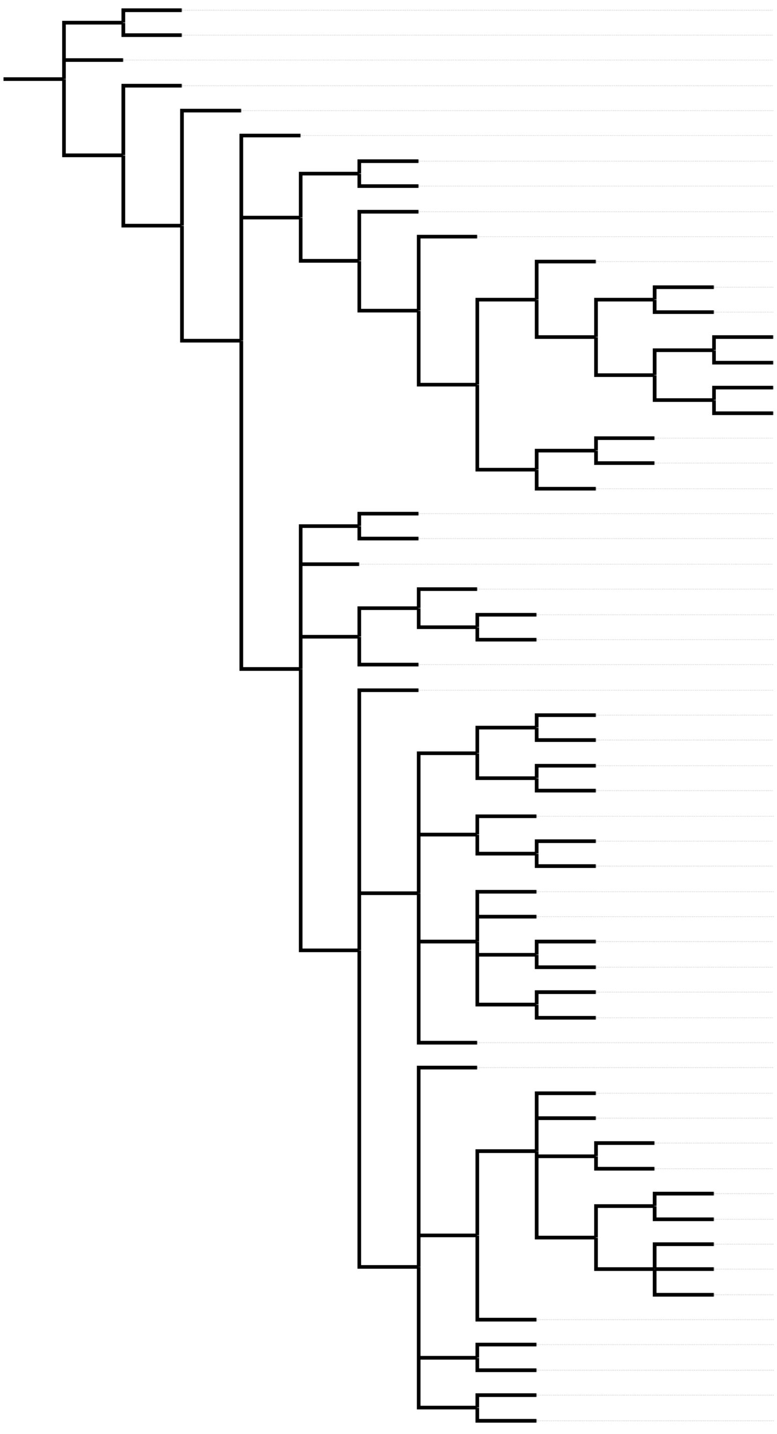

	BAHD	*SPH*	*YUC*
Sphagnum fallax	0	20	4
Physcomitrella patens	0	8	6
Marchantia polymorpha	0	3	2
Selaginella moellendorffii	0	0	3
Amborella trichopoda	7	1	6
Aquilegia coerulea	45	15	7
Spirodela polyrhiza	14	0	5
Zostera marina	11	0	7
Musa acuminata	19	0	19
Ananas comosus	20	0	8
Oropetium thomaeum	12	0	7
Zea mays	39	0	14
Sorghum bicolor	30	0	9
Panicum hallii	26	0	8
Panicum virgatum	47	0	17
Setaria viridis	30	0	8
Setaria italica	31	0	9
Brachypodium stacei	25	0	9
Brachypodium distachyon	26	0	11
Oryza sativa	19	0	8
Kalanchoe laxiflora	48	29	22
Kalanchoe fedtschenkoi	20	22	14
Amaranthus hypochondriacus	17	6	8
Erythranthe guttata	50	104	9
Solanum tuberosum	65	25	7
Solanum lycopersicum	38	17	9
Daucus carota	24	6	13
Vitis vinifera	24	1	6
Trifolium pratense	33	56	9
Medicago truncatula	76	66	15
Phaseolus vulgaris	38	66	12
Glycine max	66	25	24
Fragaria vesca	26	15	7
Prunus persica	38	45	7
Malus domestica	78	17	10
Turnera subulata	72	83	14
Linum usitatissimum	57	71	10
Ricinus communis	41	23	8
Manihot esculenta	32	28	14
Salix purpurea	75	11	11
Populus trichocarpa	86	18	11
Cucumis sativus	22	55	9
Eucalyptus grandis	32	18	15
Eutrema salsugineum	9	69	11
Boechera stricta	10	56	11
Brassica oleracea	11	20	15
Brassica rapa	19	109	18
Capsella grandiflora	7	71	11
Capsella rubella	12	84	11
Arabidopsis halleri	6	67	6
Arabidopsis lyrata	12	57	10
Arabidopsis thaliana	46	64	11
Carica papaya	16	10	6
Gossypium raimondii	37	24	18
Theobroma cacao	39	9	7
Citrus clementina	38	33	8
Citrus sinensis	20	25	7
Total	1741	1552	581

Supplemental Figure S4. NCBI generated common tree of the species used across the three analyses. Total members of the three gene families are accounted for at the bottom of the image.

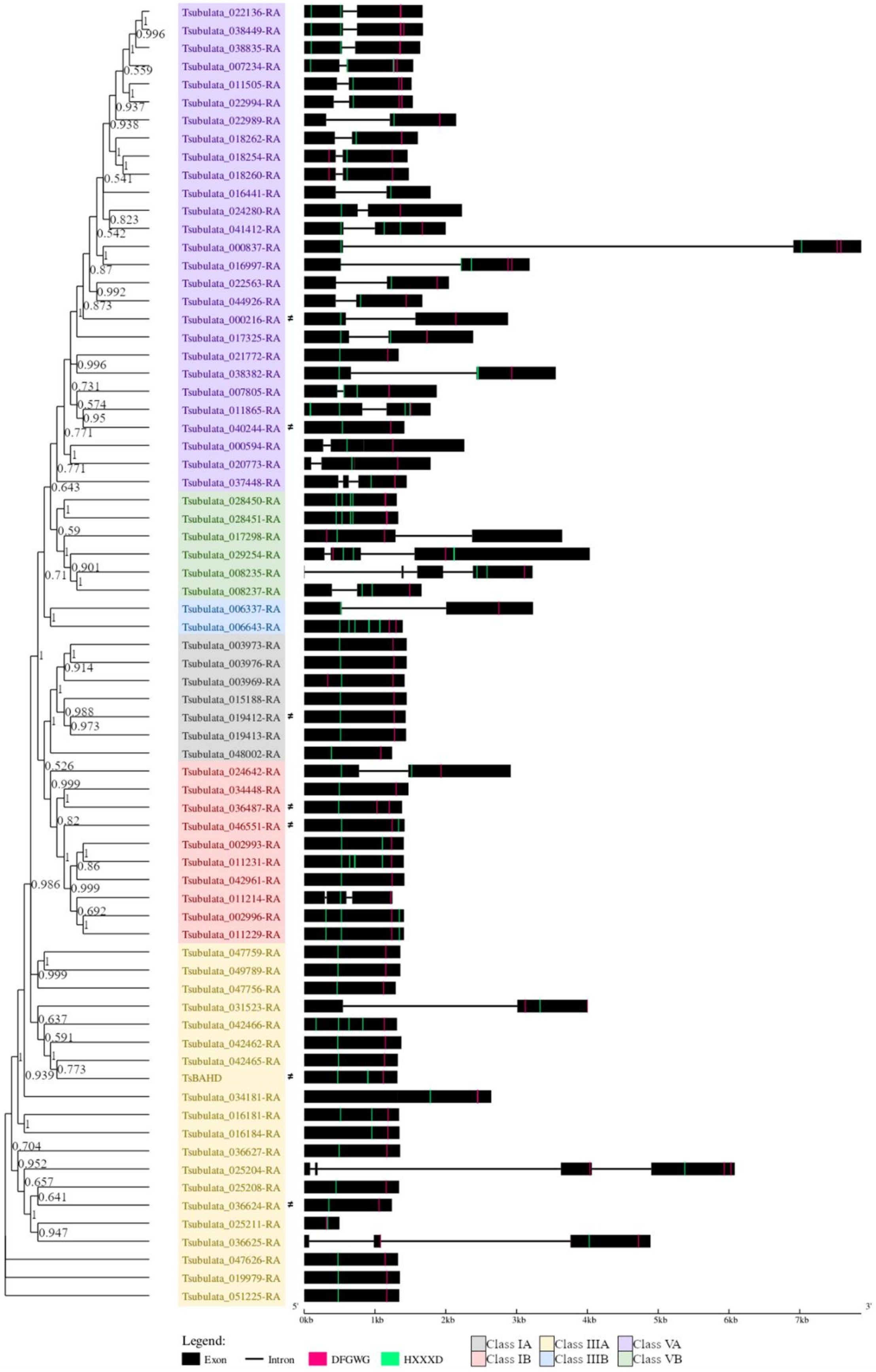
Tsubulata_022136-RA
Tsubulata_038449-RA
Tsubulata_038835-RA
Tsubulata_007234-RA
Tsubulata_011505-RA
Tsubulata_022994-RA
Tsubulata_022989-RA
Tsubulata_018262-RA
Tsubulata_018254-RA
Tsubulata_018260-RA
Tsubulata_016441-RA
Tsubulata_024280-RA
Tsubulata_041412-RA
Tsubulata_000837-RA
Tsubulata_016997-RA
Tsubulata_022563-RA
Tsubulata_044926-RA
Tsubulata_000216-RA
Tsubulata_017325-RA
Tsubulata_021772-RA
Tsubulata_038382-RA
Tsubulata_007805-RA
Tsubulata_011865-RA
Tsubulata_040244-RA
Tsubulata_000594-RA
Tsubulata_020773-RA
Tsubulata_037448-RA
Tsubulata_028450-RA
Tsubulata_028451-RA
Tsubulata_017298-RA
Tsubulata_029254-RA
Tsubulata_008235-RA
Tsubulata_008237-RA
Tsubulata_006337-RA
Tsubulata_006643-RA
Tsubulata_003973-RA
Tsubulata_003976-RA
Tsubulata_003969-RA
Tsubulata_015188-RA
Tsubulata_019412-RA
Tsubulata_019413-RA
Tsubulata_048002-RA
Tsubulata_024642-RA
Tsubulata_034448-RA
Tsubulata_036487-RA
Tsubulata_046551-RA
Tsubulata_002993-RA
Tsubulata_011231-RA
Tsubulata_042961-RA
Tsubulata_011214-RA
Tsubulata_002996-RA
Tsubulata_011229-RA
Tsubulata_047759-RA
Tsubulata_049789-RA
Tsubulata_047756-RA
Tsubulata_031523-RA
Tsubulata_042466-RA
Tsubulata_042462-RA
Tsubulata_042465-RA
TsBAHD
Tsubulata_034181-RA
Tsubulata_016181-RA
Tsubulata_016184-RA
Tsubulata_036627-RA
Tsubulata_025204-RA
Tsubulata_025208-RA
Tsubulata_036624-RA
Tsubulata_025211-RA
Tsubulata_036625-RA
Tsubulata_047626-RA
Tsubulata_019979-RA
Tsubulata_051225-RA
5'
3'
0kb 1kb 2kb 3kb 4kb 5kb 6kb 7kb
Legend:
Exon
Intron
DFGWG
HXXXD
Class IA
Class IB
Class IIIA
Class IIIB
Class VA
Class VB

Supplemental Figure S6. Phylogenetic relationship of the putative *BAHD* family members *Turnera subulata*. ≠ marks genes that were previously identified as differentially expressed [16]. A comprehensive list of differentially expressed *BAHD*s can be found in Supplementary Table S2. Figure was generated using GSDS (V-2.0) [109]

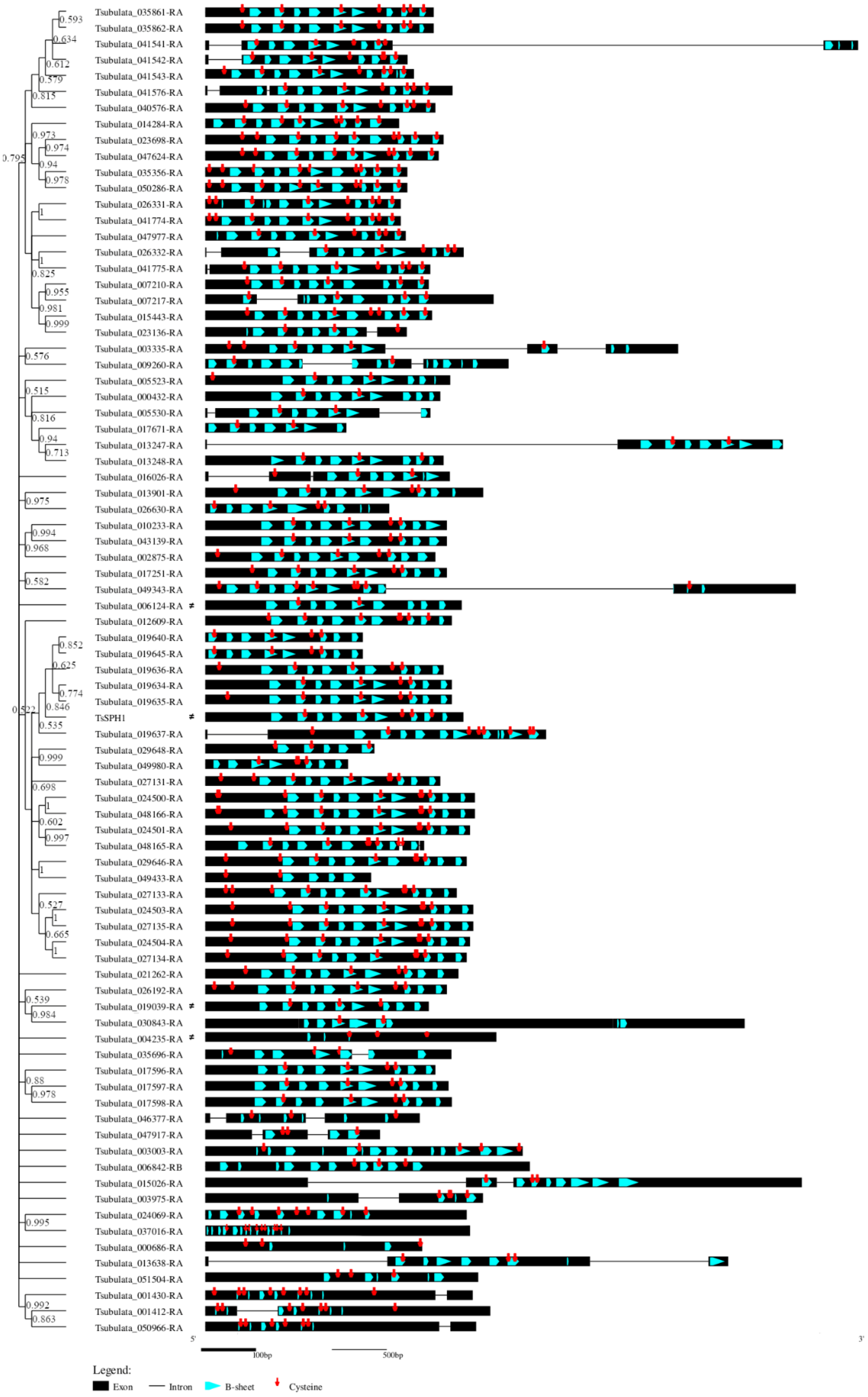
Tsubulata_035861-RA
Tsubulata_035862-RA
Tsubulata_041541-RA
Tsubulata_041542-RA
Tsubulata_041543-RA
Tsubulata_041576-RA
Tsubulata_040576-RA
Tsubulata_014284-RA
Tsubulata_023698-RA
Tsubulata_047624-RA
Tsubulata_035356-RA
Tsubulata_050286-RA
Tsubulata_026331-RA
Tsubulata_041774-RA
Tsubulata_047977-RA
Tsubulata_026332-RA
Tsubulata_041775-RA
Tsubulata_007210-RA
Tsubulata_007217-RA
Tsubulata_015443-RA
Tsubulata_023136-RA
Tsubulata_003335-RA
Tsubulata_009260-RA
Tsubulata_005523-RA
Tsubulata_000432-RA
Tsubulata_005530-RA
Tsubulata_017671-RA
Tsubulata_013247-RA
Tsubulata_013248-RA
Tsubulata_016026-RA
Tsubulata_013901-RA
Tsubulata_026630-RA
Tsubulata_010233-RA
Tsubulata_043139-RA
Tsubulata_002875-RA
Tsubulata_017251-RA
Tsubulata_049343-RA
Tsubulata_006124-RA ≠
Tsubulata_012609-RA
Tsubulata_019640-RA
Tsubulata_019645-RA
Tsubulata_019636-RA
Tsubulata_019634-RA
Tsubulata_019635-RA
TsSPH1 ≠
Tsubulata_019637-RA
Tsubulata_029648-RA
Tsubulata_049980-RA
Tsubulata_027131-RA
Tsubulata_024500-RA
Tsubulata_048166-RA
Tsubulata_024501-RA
Tsubulata_048165-RA
Tsubulata_029646-RA
Tsubulata_049433-RA
Tsubulata_027133-RA
Tsubulata_024503-RA
Tsubulata_027135-RA
Tsubulata_024504-RA
Tsubulata_027134-RA
Tsubulata_021262-RA
Tsubulata_026192-RA
Tsubulata_019039-RA ≠
Tsubulata_030843-RA
Tsubulata_004235-RA ≠
Tsubulata_035696-RA
Tsubulata_017596-RA
Tsubulata_017597-RA
Tsubulata_017598-RA
Tsubulata_046377-RA
Tsubulata_047917-RA
Tsubulata_003003-RA
Tsubulata_006842-RB
Tsubulata_015026-RA
Tsubulata_003975-RA
Tsubulata_024069-RA
Tsubulata_037016-RA
Tsubulata_000686-RA
Tsubulata_013638-RA
Tsubulata_051504-RA
Tsubulata_001430-RA
Tsubulata_001412-RA
Tsubulata_050966-RA
5'
3'
100bp
500bp
Legend:
Exon
Intron
B-sheet
Cysteine

Supplemental Figure S8. Phylogenetic relationship of the putative *SPH* family members in *Turnera subulata.* ≠ marks genes that were previously identified as differentially expressed [16]. A comprehensive list of differentially expressed *SPH*s can be found in Supplementary Table S2. Figure was generated using GSDS (v.2.0) [109].

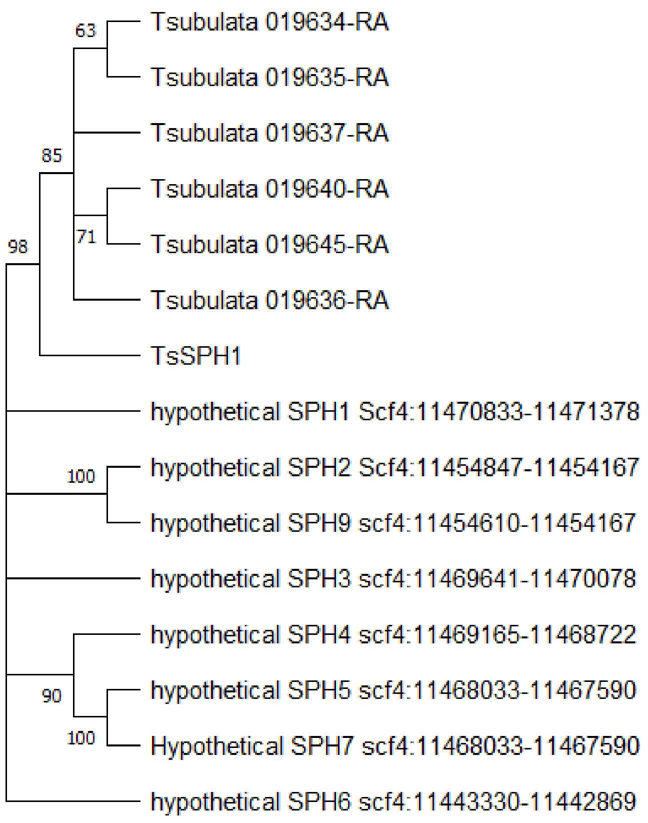

Supplemental Figure S9. Phylogenetic analysis of the *Passiflora organensis* and *Turnera subulata SPH*s. These represent orthologs that hypothetically reside on an orthologous chromosome. *Turnera subulata SPH*s can be found on scaffold 850_149123; *Passiflora organensis SPH*s can be found on scaffold 4. Nomenclature of *Passiflora organensis* genes was arbitrarily assigned. Generated using MEGA11 [30]. Values at nodes represent proportions from 1000 bootstrap replicates.

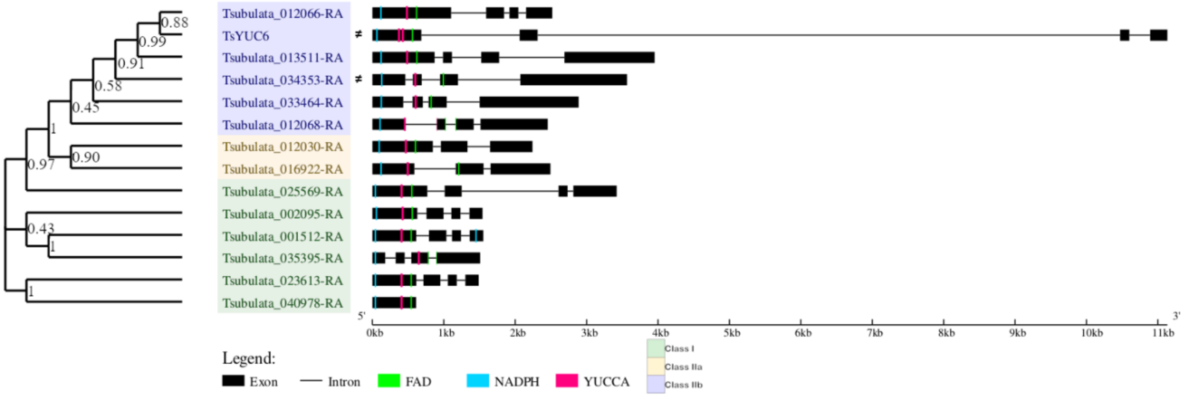

Supplemental Figure S11. Phylogenetic relationship of the putative *YUCCA* family members in *Turnera subulata*. ≠ marks genes that were previously identified as differentially expressed [16]. A comprehensive list of differentially expressed *YUCCA*s can be found in Supplementary Table S2. Figure was generated using GSDS (v.2.0) [109]

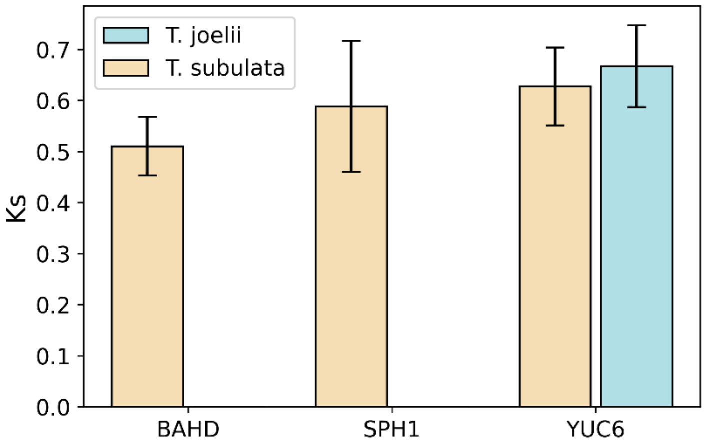

Supplemental Figure S12. Synonymous substitution rates (Ks) for the three *S*-genes relative to their closest paralogs (*TsBAHD* vs Tsubulata_042462-RA; *TsSPH1* vs Tsubulata_19640-RA; *TsYUC6* vs Tsubulata_012066-RA; *TjYUC6* vs OasesvelvLoc16468t6 (GenBank: OP886701) as calculated using methods outlined in Gutiérrez-Valencia *et al*., 2022. Error bars represent the standard errors.

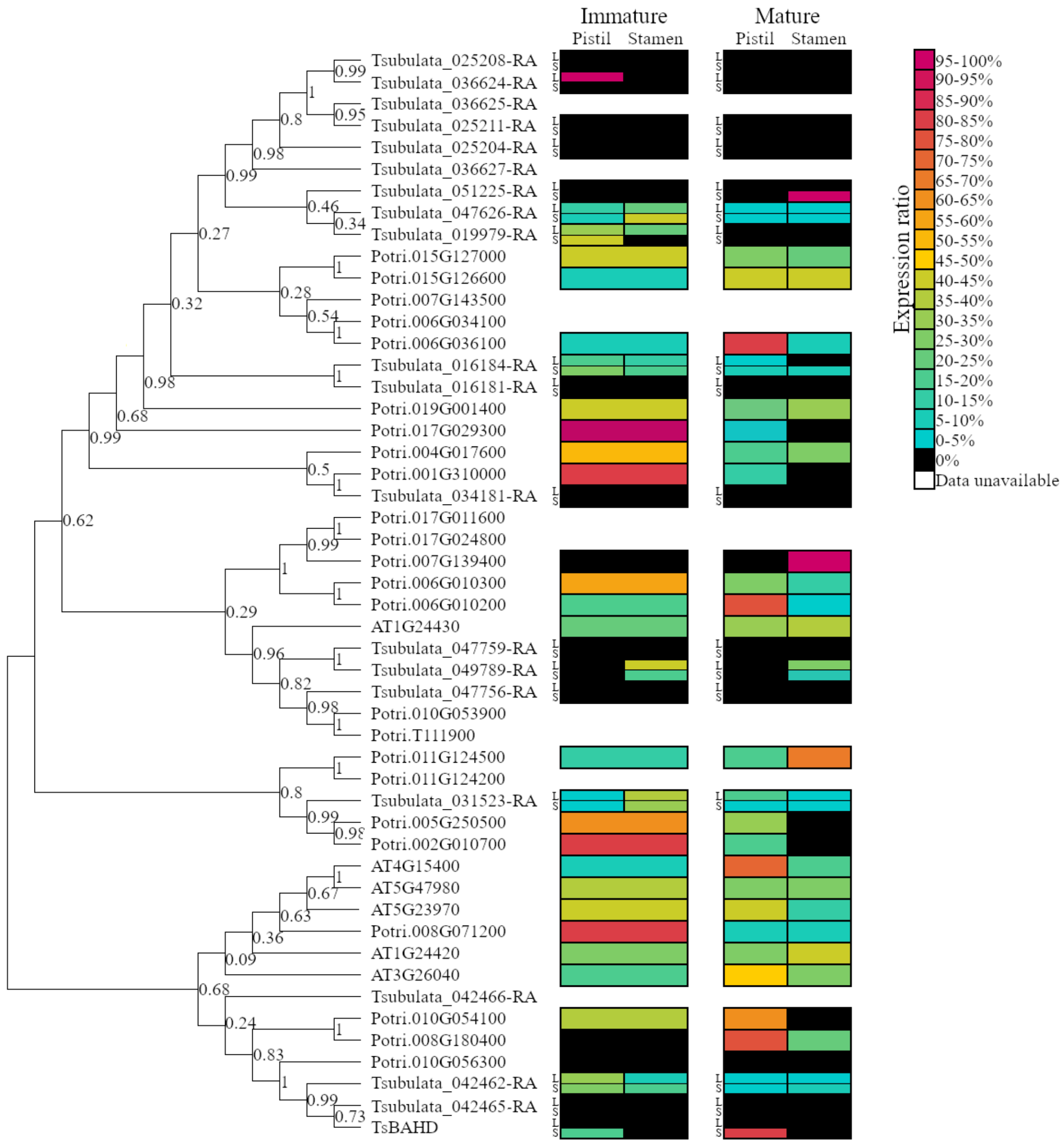

Supplementary Figure S13: Comparison of the expression of the IIIA subclade of the *BAHD* family in the developing flowers of *Arabidopsis* (stage 9, stage 12 carpel and stamen), *Populus* (samples BESC423.ZL, GW9592.ZK, and Early dormant bud), and *Turnera* (PRJNA589060). Tree represents the maximum likelihood estimate of relationship including bootstrap values. Expression patterns were normalized within species but not across species. Expression patterns were normalized for individual genes not across genes.

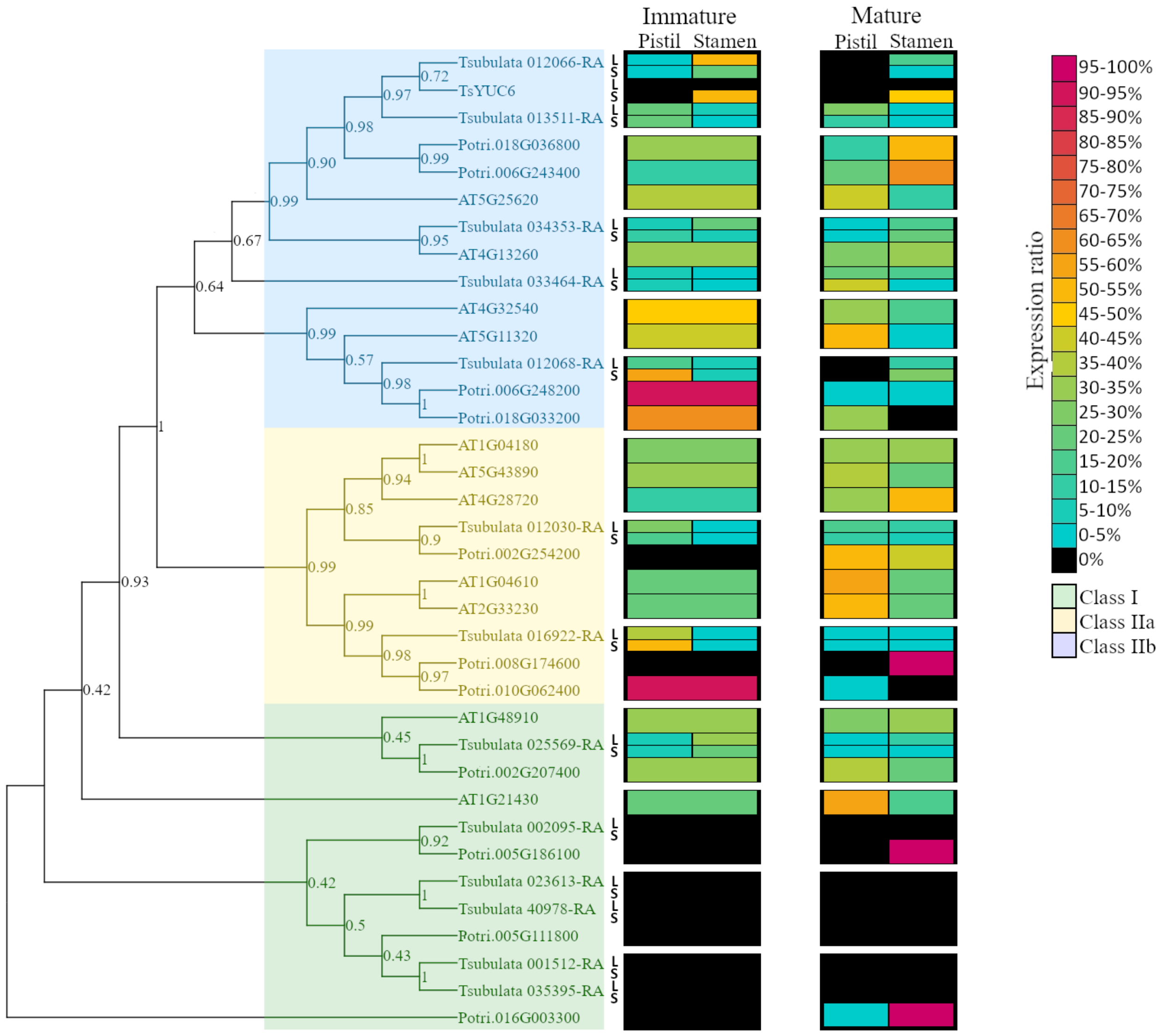

Supplementary Figure S14: Comparison of the expression of the *YUCCA* family in the developing flowers of *Arabidopsis*, *Populus*, and *Turnera*. Expression patterns were normalized for individual genes not across genes.

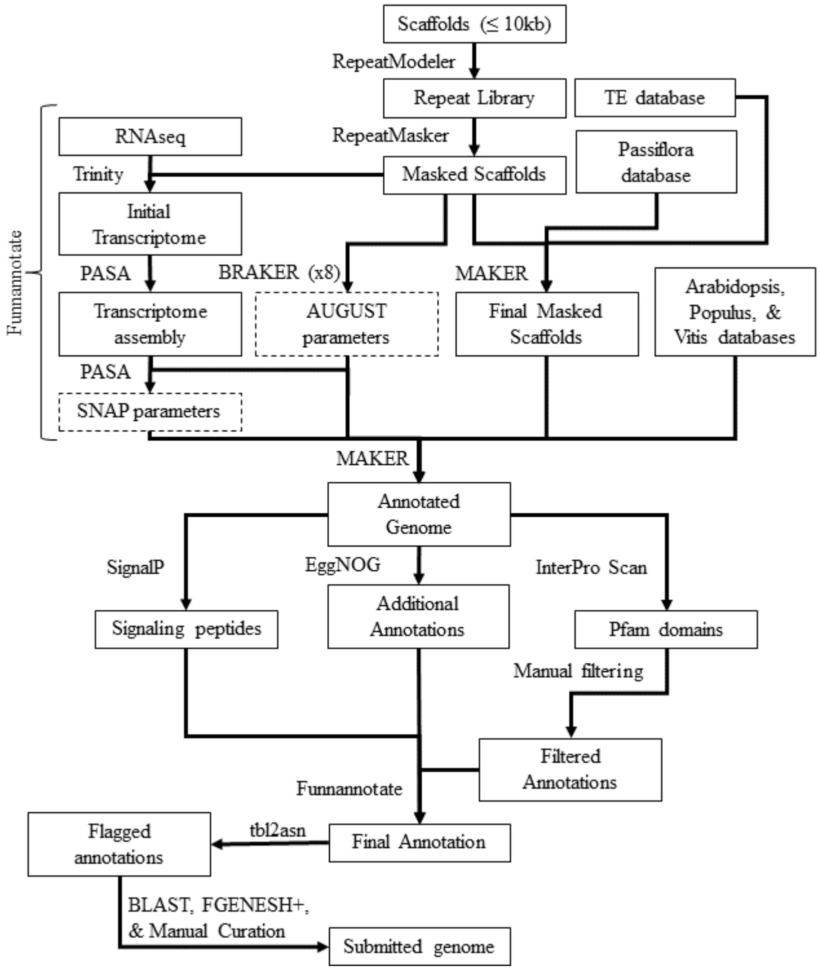

Supplementary Figure S15. Pipeline used for genome annotation. Solid boxes represent inputs and outputs. Broken boxes represent generated parameters
