# Supplementary figures and images for: Annotation of the Turnera subulata (Passifloraceae) Draft Genome Reveals the S-Locus Evolved after the Divergence of Turneroideae from Passifloroideae in a Stepwise Manner
Source: Plants (Basel). 2023 Jan 7;12(2):286. doi: 10.3390/plants12020286 (PMC9862265; doi:10.3390/plants12020286)

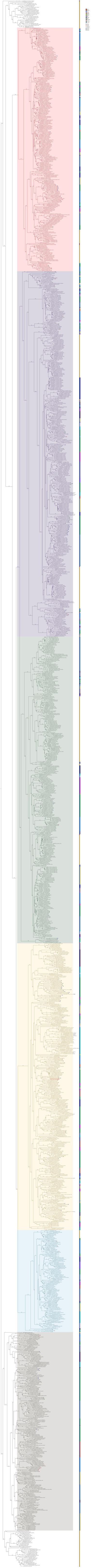

Supplement: Supplementary file 1 [file plants-12-00286-s001.zip › plants-2098363-supplementary (2)/Supplementary_Figure S5.pdf]
