# Supplementary material for: Annotation of the Turnera subulata (Passifloraceae) Draft Genome Reveals the S-Locus Evolved after the Divergence of Turneroideae from Passifloroideae in a Stepwise Manner
Source: Plants (Basel). 2023 Jan 7;12(2):286. doi: 10.3390/plants12020286 (PMC9862265; doi:10.3390/plants12020286)
Supplement: Supplementary file 1 [file Supplementary_Figure S12.pdf › plants-2098363-supplementary (2)/Supplementary_Figure S12.pdf]

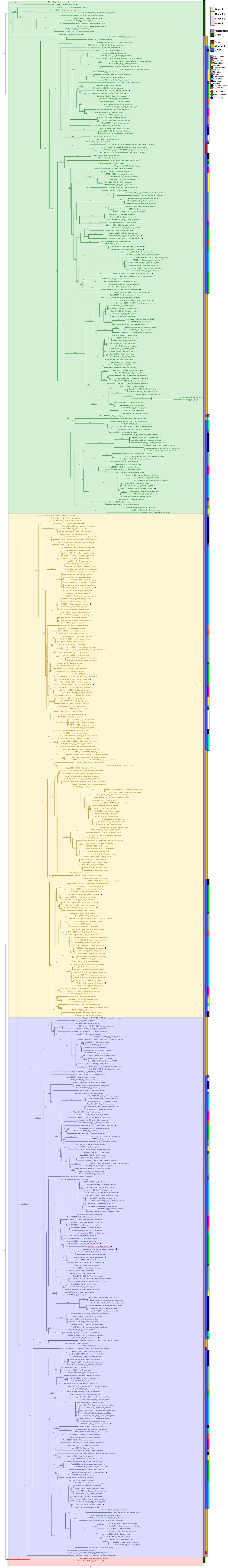

Class I
Class IIa
Class IIb
Class II
Angiosperm
Other
Other
Monocot
Dicot
Brassicales
Malvales
Sapindales
Malpighiales
Rosales
Cucurbitales
Fabales
Myrtales
Vitales
Gentianales
Solanales
Lamiales
Apiales
Caryophyllales
Ranunculales
A. thaliana
P. trichocarpa
T. subulata
